# Wild *Prunus cerasifera* Ehrh. Polyphenols Alleviate Hyperglycemia in Type 2 Diabetes Mellitus Mice via Modulating Gut Microbiota‐SCFAs‐PI3K/Akt/TBC1D4 Pathway

**DOI:** 10.1002/fsn3.71498

**Published:** 2026-02-08

**Authors:** Xinpeng Cheng, Xing Xie, Shibo Luo, Quanyuan Xie, Haiyan Xiang, Chunyan Peng, Qiao Ding, Hongbing Fan, Wei Liu, Lu Zhang

**Affiliations:** ^1^ National R&D Center of Freshwater Fish Processing, College of Life Science Jiangxi Normal University Nanchang Jiangxi China; ^2^ Key Lab of Natural Product Chemistry and Application at Universities of Education Department of Xinjiang Uygur Autonomous Region, School of Chemistry and Chemical Engineering Yili Normal University Yining China; ^3^ School of College Jiangxi University of Technology Nanchang China; ^4^ Department of Animal and Food Sciences University of Kentucky Lexington Kentucky USA

**Keywords:** glucose metabolism, gut‐liver axis, phenolics, wild *Prunus cerasifera* Ehrh.

## Abstract

Our previous work demonstrated that wild *Prunus cerasifera* Ehrh. possesses notable in vitro hypoglycemic activity. This study further evaluated the effects of wild *Prunus cerasifera* Ehrh. polyphenol extract (WPPE) on T2DM mice. WPPE treatment significantly lowered blood glucose, improved insulin resistance, and reduced hepatic oxidative stress and inflammation in T2DM mice. It could promote the production of short‐chain fatty acids and regulate the relative abundance of T2DM‐associated bacteria like *Muribaculaceae* and *Odoribacter*, and enhance hepatic glycogen synthesis through activation of the key gene involved in the PI3K/AKT/TBC1D4 pathway, resulting in hypoglycemic effect based on the gut‐liver axis. These findings support WPPE as a promising dietary candidate for T2DM management.

## Graphical abstract

WPPE treatment significantly alleviated type 2 diabetes by improving SCFAs and gut microbiota composition and promoted glycogen synthesis by regulating the PI3K/AKT/TBC1D4 pathway. WPPE also improved liver function and reduced oxidative stress and inflammation.

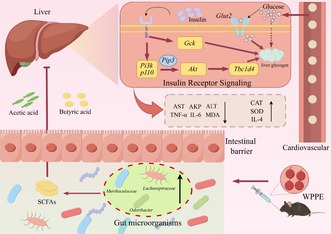

**Abbreviations**
AKP: alkaline phosphatase
ALT: alanine aminotransferase
AST: aspartate aminotransferase
CAT: catalase
DEGs: differentially expressed genes
F/B: firmicutes/bacteroidetes
FBG: fasting blood glucose
GSP: glycosylated serum protein
H&E: hematoxylin and eosin
HbA1c: glycosylated hemoglobin, type A1C
HOMA‐IR: homeostatic model assessment of insulin resistance
IL‐4: interleukin‐4
IL‐6: interleukin‐6
IR: insulin resistance
MDA: malondialdehyde
SCFAs: short‐chain fatty acids
SOD: superoxide dismutase
STZ: streptozotocin
TC: total cholesterol
TG: triglyceride
TNF‐α: tumor necrosis factor‐alpha
WPPE: wild *Prunus cerasifera* polyphenol extract

## 1 Introduction

Type 2 diabetes mellitus (T2DM) is a chronic metabolic disease characterized by sustained hyperglycemia. According to the latest statistics, approximately 589 million people worldwide were diagnosed with diabetes in 2025, among whom 252 million were unaware of their condition (Colagiuri and Ceriello 2025). Prolonged hyperglycemia stimulates the production of reactive oxygen species and advanced glycation end products within the body, thereby significantly increasing the incidence of diabetic complications, particularly cardiovascular and renal diseases (Cole and Florez 2020). Commonly prescribed antidiabetic drugs include sulfonylureas, glinides, and acarbose, which is often accompanied by potential side effects, such as digestive discomfort, fluid retention, and skin hypersensitivity (Dahlén et al. 2021). Dietary intervention through food nutritional ingredients has been considered an effective approach for preventing T2DM. Therefore, scientific investigations have increasingly focused on exploring bioactive compounds with antidiabetic properties, particularly phytochemicals from edible plant species, due to their combined nutritional and therapeutic potential.

Accumulating evidence demonstrates that gut microbiota are intimately related to the development of T2DM and can be positively influenced by the intake of certain phytochemicals (Blandino et al. 2016). For instance, short‐chain fatty acids (SCFAs) can effectively reduce blood glucose levels and improve insulin resistance (IR) (Priyadarshini et al. 2016). They can be transported to the liver through veins and then relieve hepatic inflammation and promote hepatic glycogen synthesis by modulating key intracellular signaling pathways like PI3K/AKT and AMPK (Bauer et al. 2022; Jiang et al. 2023). Dietary polyphenols effectively ameliorate T2DM‐induced intestinal metabolic disorders and dysregulation of liver signaling pathways. For example, sweet potato leaf polyphenols demonstrated significant therapeutic potential in ameliorating insulin resistance and enhancing glucose homeostasis through targeted modulation of the hepatic PI3K/AKT/GSK‐3β signaling pathway in type 2 diabetic mice models. Besides, phenolic compounds derived from highland barley may ameliorate hyperlipidemia by regulating gut microbiota composition and AMPK pathways (Deng et al. 2020). Moreover, polyphenols have low bioavailability and are unstable. The gut microbiota can enhance the bioavailability of polyphenols, while polyphenols, in turn, help maintain the integrity of the intestinal barrier and the homeostasis of the gut microbiota (Zhou et al. 2020). Therefore, restoring the balance of gut microbiota and SCFAs has emerged as an effective strategy for preventing and treating T2DM, and prebiotic phenolics from edible foods show great therapeutic potential.

Wild cherry plum ( *Prunus cerasifera* Ehrh.) is a shrub belonging to the *Rosaceae* family, native to Asia and eastern/central Europe, which is widely distributed in Huocheng County, Xinjiang, China (Wang et al. 2012). Wild cherry plum fruit is often processed into jams, juices, and fruit wines. It is rich in various phenolics, including flavonoids, phenolic acids, anthocyanins, etc. (Cevallos‐Casals et al. 2006), which is the best resource of active ingredients. Moruzzi et al. (2021) discovered that *Prunus cerasus* L. extract could reduce inflammatory markers levels like tumor necrosis factor alpha (TNF‐α) and interleukin‐1 beta (IL‐1 β) in obese rats. Our previous study found that wild cherry plum fruit extract exhibited strong in vitro α‐glucosidase and dipeptidyl peptidase‐4 inhibitory activities, and its major phenolic constituents included caffeoylquinic acid, apigenin, quercetin‐3‐*O*‐glucoside, and protocatechuic acid (Luo et al. 2024). In short, the current research in cherry plum polyphenols was focused on biological activity, such as antioxidant and anti‐inflammatory properties, but the regulatory mechanisms are still unclear. (Gündüz and Saraçoğlu 2012; Saraswathi et al. 2020) Although WPPE exhibited excellent in vitro hypoglycemic activity, while the in vivo hypoglycemic mechanism of it remains to be elucidated, especially for the modulation effect on key targets of diabetes mellitus in gut‐liver axis like gut microbiota, SCFAs and PI3K/Akt pathway.

The aim of this study was to investigate the in vivo hypoglycemic effect of WPPE and its underlying mechanisms based on the gut microbiota‐SCFAs‐hepatic signaling pathway axis. The T2DM mice were used as a model, and the effect of WPPE on the basic biochemical indicators was evaluated, and the key gut microbiota, SCFAs, and related hepatic signaling pathway regulated by WPPE were further screened through metabolomics, transcriptomics, and gas chromatography–mass spectrometry (GC–MS) technologies. This study provides new evidence for supporting the potential of wild cherry plum fruit as dietary supplements for T2DM management.

## 2 Materials and Methods

### 2.1 Reagents and Materials

Wild *Prunus cerasifera* was collected from Huocheng County, Xinjiang Uyghur Autonomous Region. The commercial test and enzyme‐linked immunosorbent assay (ELISA) kits were respectively purchased from Jiancheng Bioengineering Institute (Nanjing, China) and Biyuntian Biotechnology (Shanghai, China), and the detailed information was listed in Table S1. Antibodies were purchased from Takara Bio Inc. (Beijing, China). Other chemical reagents were purchased from Shanghai Yuanye Biotechnology (Shanghai, China).

### 2.2 Preparation of Wild *Prunus cerasifera* Polyphenols

Wild *Prunus cerasifera* was ground into powder and was extracted with 70% ethanol aqueous solution (1:20, w/v) at room temperature for 2 h. After filtration, the residue was extracted twice under the same conditions. All supernatants were collected and concentrated to get *Prunus cerasifera* extract (PCE). PCE was dissolved with distilled water and sequentially extracted with 10 volumes of petroleum ether and ethyl acetate. The wild *Prunus cerasifera* polyphenol extract (WPPE) was obtained by concentrating and evaporating the ethyl acetate fraction under vacuum and was stored at −20°C for further analysis. The chemical composition of WPPE consisted of total phenolics (219.27 mg GAE/g E), total flavonoids (165.58 mg QuE/g E) (Luo et al. 2024), total sugar (326.61 μg GLU/mg E), total proteins (75.20 μg BSA/mg E), and ash (0.34%) (shown in Table S2).

### 2.3 Animal Experimental Design

**FIGURE 1:**
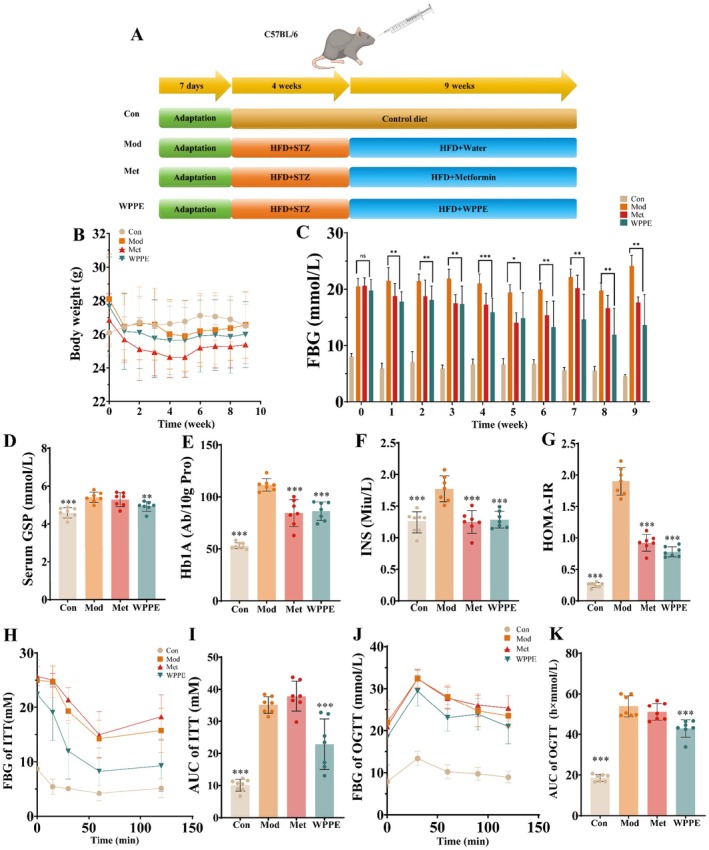
Hypoglycemic effects of WPPE supplementation on T2D mice. (A) Experimental design scheme. (B) Body weight. (C) Fasting blood glucose. (D) Serum GSP content. (E) Hb1A content. (F) Insulin level. (G) HOMA ‐ IR. (H) FBG of ITT test. (I) AUC value of ITT. (J) FBG of OGTT test. (K) AUC value of OGTT test. *p < 0.05, **p < 0.01, and ***p < 0.001 represented the significant difference of other groups with Mod group.

The animal experimental was carried out in accordance with the guidelines of Ethics Committee of Nanchang Research Institute in Sun Yat‐sen University (SYSUNC‐IACUC‐B‐2025‐0001). Fifty C57BL/6J mice (15 ± 1 g) were obtained from Charles River Laboratories (Beijing, China) and were housed for 1 week at a temperature of 25°C ± 2°C, humidity of 50% ± 5% with a 12 h day/night cycle. All mice were assigned randomly into two groups; normal diet group was given a normal chow (D12451) (*n* = 7), and high‐fat group was given a high‐fat diet (HFD) (D12450B) (*n* = 43). After 30 days of feeding, then HFD mice were fasted for 12 h and injected with streptozotocin (STZ) solution (dissolved in pH 4.5, 0.1 mmol/L citrate buffer) at a dose of 100 mg/kg body weight for once after 4 weeks. Following a 7‐day experimental period, fasting blood glucose (FBG) levels were measured; individuals with FBG > 11.1 mmol/L were classified as T2DM models. The 21 successfully modeled T2DM mice were selected and randomly divided into three groups (*n* = 7) by Rv.Uniform function in SPSS software: model group (Mod), metformin group (Met), and WPPE group. Mice that failed to develop hyperglycemia after STZ induction were euthanized and excluded from further experiments. The design was illustrated in Figure 1A. The Met200 and WPPE groups were treated with 200 mg/kg/day of Met and WPPE, respectively, while the Mod and Con groups were treated with equivalent of saline solution for 9 weeks. Meanwhile, the body weight and FBG level of mice were detected every week. At the conclusion of the experiment, all mice were euthanized, and serum was isolated by centrifuging blood samples at 3000 rpm for 10 min. Meanwhile, the liver, pancreas, and colon tissues were collected and stored at −80°C for further analysis.

### 2.4 Oral Glucose Tolerance Test (OGTT) and Insulin Tolerance Test (ITT)

After supplementation of samples for 8 weeks, all mice were fasted for 12 h before oral gavage of 1.0 g/kg body weight of glucose or intraperitoneally administration of 0.75 U/kg body weight of insulin. The blood glucose levels of all mice were respectively evaluated at 0, 30, 60, 90, and 120 min after injection. The area under the curve (AUC) was calculated using Origin 9.0 software, and the formula was as follows:

$$\mathrm{AUC}\;\text{value}=\sum \left(\frac{y_{i}+y_{i+1}}{2}\times \left(x_{i+1}-x_{i}\right)\right)$$

where $y_{i}$ and $y_{i+1}$ are the blood glucose or insulin level at different time points, and $x_{i}$ and $x_{i+1}$ are the corresponding time points.

### 2.5 Biochemical and Histological Assay

The serum concentrations of insulin, glycosylated serum protein (GSP), hemoglobin A1c (Hb1A), alanine aminotransferase (ALT), aspartate aminotransferase (AST), and alkaline phosphatase (AKP) were evaluated using commercial kits. The hepatic levels of catalase (CAT), superoxide dismutase (SOD), malondialdehyde (MDA), tumor necrosis factor‐alpha (TNF‐α), interleukin‐6 (IL‐6), and interleukin‐4 (IL‐4) were determined by corresponding commercial kits according to the instructions. The homeostatic model assessment of insulin resistance (HOMA‐IR) was analyzed as follows:

(1) $$\text{HOMA}-\mathrm{IR}=\left[\text{fasting insulin level}\;\left(\mathrm{mU}/L\right)\times \mathrm{FBG}\;\text{level}\;\left(\text{mmol}/L\right)\right]/22.5$$

Histological analysis of liver was conducted by Oil Red O staining. In brief, the liver tissues were fixed in 4% neutral formalin solution, and then dehydrated, embedded, sectioned, and stained with hematoxylin and eosin (H&E) as well as Oil Red O. Finally, the samples were examined using a Nikon Eclipse E100 Upright Optical Microscope (Nikon Corporation, Tokyo, Japan) to obtain detailed morphological observation.

### 2.6 Hepatic Transcriptome Analysis

Total RNA was extracted from liver by using TRIzol reagent (Invitrogen, Carlsbad, CA, USA) according to the instructions. Agarose gel electrophoresis was performed to analyze the integrity of RNA and eliminate DNA contamination. The library insert size was checked using a fragment analyzer. The mRNA (mRNA) was enriched with immobilized oligo (dT) primers and fragmented, then cDNA was synthesized for building pair‐ended (PE) RNA‐seq libraries. After passing the library inspection, the libraries were sequenced using the Illumina platform (San Diego, CA). The raw sequencing data was filtered by FASTP software, and mainly included the following parts: (1) Read data with adapters; (2) Discard paired reads with more than 10% N content; (3) Remove paired reads with over 50% low‐quality bases (*Q* ≤ 20). And the clean reads were compared with the reference genome using HISAT2 software. Gene expression levels were quantified using the FPKM method, and differentially expressed genes (DEGs) were screened using the criteria of *p* < 0.05, FDR < 0.05 and |log_2_Fold Change| ≥ 1. Function annotation of DEGs was obtained by finding the Kyoto Encyclopedia of Genes and Genomes (KEGG) databases, and KEGG pathway enrichment analysis was performed by KOBAS software.

### 2.7 RT‐qPCR Analysis

The key target genes screened from the hepatic transcriptome analysis were quantified using RT‐qPCR. The total RNA was extracted from liver tissue using Trizol reagent and quantified by a NanoDrop‐1000 spectrophotometer (Wilmington, DE, USA). Subsequently, RNA was reverse transcribed into cDNA using the PrimeScript RT Reagent Kit. RT‐qPCR was performed to quantify the relative expression of key target genes, including phosphoinositide 3‐kinase (*Pi3k*), protein kinase B (*Akt*), TBC1 domain family member 4 (*Tbc1d4*), glucokinase (*Gck*), and glucose transporter type 2 (*Glut2*). The glyceraldehyde‐3‐phosphate dehydrogenase (*Gapdh*) was selected as an internal control gene, and primer efficiencies were confirmed to be within the acceptable range (90%–110%). The detailed primer information was listed in Table S3. The ΔΔC_t_ calculation formula was as follows, and Ct represented the cycle threshold value.

(2) $$\Delta C_{t}=C_{t}\;\left(\text{Target gene}\right)-C_{t}\;\left(\text{Internal control gene}\right)$$

(3) $$\text{Relative expression level}=2^{-\Delta \Delta C_{t}}=2^{-\left(\Delta C_{t}\left(\text{Experimental group}\right)-\Delta C_{t}\left(\text{Control group}\right)\right)}$$

### 2.8 Short‐Chain Fatty Acids (SCFAs) Analysis

After oral administration for 9 weeks, fecal samples were collected and immediately stored at −80°C. One hundred and fifty milligrams of fecal samples were mixed with 500 μL of methyl tert‐butyl ether and were ground for 10 min. Then, 2% concentrated hydrochloric acid was added, the samples were extracted in an ice bath for 10 min. After centrifugation at 12,000 rpm for 10 min at 4°C, the supernatants were obtained and filtered through a 0.22 μm organic membrane, which was analyzed using 8890GC system equipped with an agilent HP‐FFAP column (30 m × 0.25 mm, 0.25 μm; Agilent J&W Scientific, Folsom, CA, USA). The calibration curves, linear range and *R* ^2^ values of the SCFAs standards were presented in Table S4.

### 2.9 Gut Microbiota Analysis

DNA of fecal sample was extracted using E.Z.N.A. Soil DNA Kit (Omega Bio‐Tek, Norcross, GA, USA) according to the manufacturer's instructions. The V3–V4 regions of bacterial 16S rDNA were amplified by polymerase chain reaction (PCR) with primers 338F and 806R. The amplicons were sequenced using the Illumina Miseq PE300 platform. Raw reads were trimmed and assembled, and the sequence data were classed into respective samples according to barcodes. The clustering sequences with 97% similarity were considered operational taxonomic units (OTUs). The α and β diversities were analyzed using the QIIME2 software. All samples were rarefied to 15,000 reads based on coverage curves, and the rarefaction curve was displayed in the Figure S2.

### 2.10 Statistical Analysis

All data were expressed as mean ± standard deviation (SD). Statistical analysis was performed using one‐way ANOVA followed by Tukey's *post hoc* test. Significant differences were determined when the *p* value was < 0.05 (*p < 0.05, **p < 0.01, ***p < 0.001). Spearman's correlation analysis was used to explore the relationship among gut microbiota, key target genes and basic indicators.

## 3 Results

### 3.1 WPPE Improved Glucose and Lipid Metabolism Disorders in T2DM Mice

As shown in Figure 1B, no significant changes in body weight (*p* > 0.05) were observed among the groups after 9 weeks of WPPE treatment, suggesting that WPPE did not influence weight gain in T2DM mice. As displayed in Figure 1C, the initial blood glucose levels of all experiment groups were comparable (*p* > 0.05). After 9 weeks of supplementation, the blood glucose levels in T2DM mice treated with WPPE was significantly reduced to 10.5 mmol/L (*p* < 0.05), and no significant difference was detected between WPPE and Met groups (*p* > 0.05). From Figure 1D,E, in comparison to the Mod group, WPPE intervention exhibited significant reduction of 0.48 mmol/L and 25.27 Ab/10 g protein in GSP and Type A1C glycosylated hemoglobin (HbA1c) levels, respectively (*p* < 0.05). The findings demonstrated that WPPE showed a great glucose‐lowering effect.

Figure 1F–K showed the effect of WPPE treatment on IR. Compared to the Con group, insulin levels in the model group displayed a marked increase (*p* < 0.05), whereas WPPE intervention significantly attenuated the elevation (*p* < 0.001) (Figure 1F). The HOMA‐IR index in WPPE group was also reduced by 58.7% by compared with the Mod group (Figure 1G). Further, no significant difference was observed between WPPE and Met groups (*p* > 0.05). For ITT test, the blood glucose level of T2DM mice was reduced after intraperitoneal injection of insulin, while the decline of WPPE group was obviously faster than that of Mod group (Figure 1H). During the OGTT, T2DM mice exhibited a rapid elevation in blood glucose concentration, peaking at 30 min post‐administration, and followed by a delayed glucose clearance rate (Figure 1J). Similarly, the blood glucose level in WPPE treatment group was significantly lower than Mod group throughout the experimental period. The AUC serves as a quantitative indicator of glucose tolerance in mice. The control group exhibited a significantly lower AUC value compared to the model group (*p* < 0.05). Notably, WPPE intervention effectively improved the pathological elevation of AUC in T2DM mice (*p* < 0.05). In comparison with Mod group, the AUC values for ITT and OGTT tests in WPPE group were respectively reduced by 34.85% and 8.9%, respectively. As shown in Figure S1, in comparsion with Mod group, the serum levels of TC, TG, and LDL‐C in WPPE group were decreased by 1.1, 0.43, and 1.79 mmol/L, while the HDL‐C level in was increased by 2.25 mmol/L. The above results suggested that WPPE could enhance glucose tolerance, relieve IR and reduce lipid accumulation, thus modulated glucose and lipid metabolism of T2DM.

### 3.2 WPPE Alleviated Hepatic Oxidative Stress and Inflammation Caused by T2DM

**FIGURE 2:**
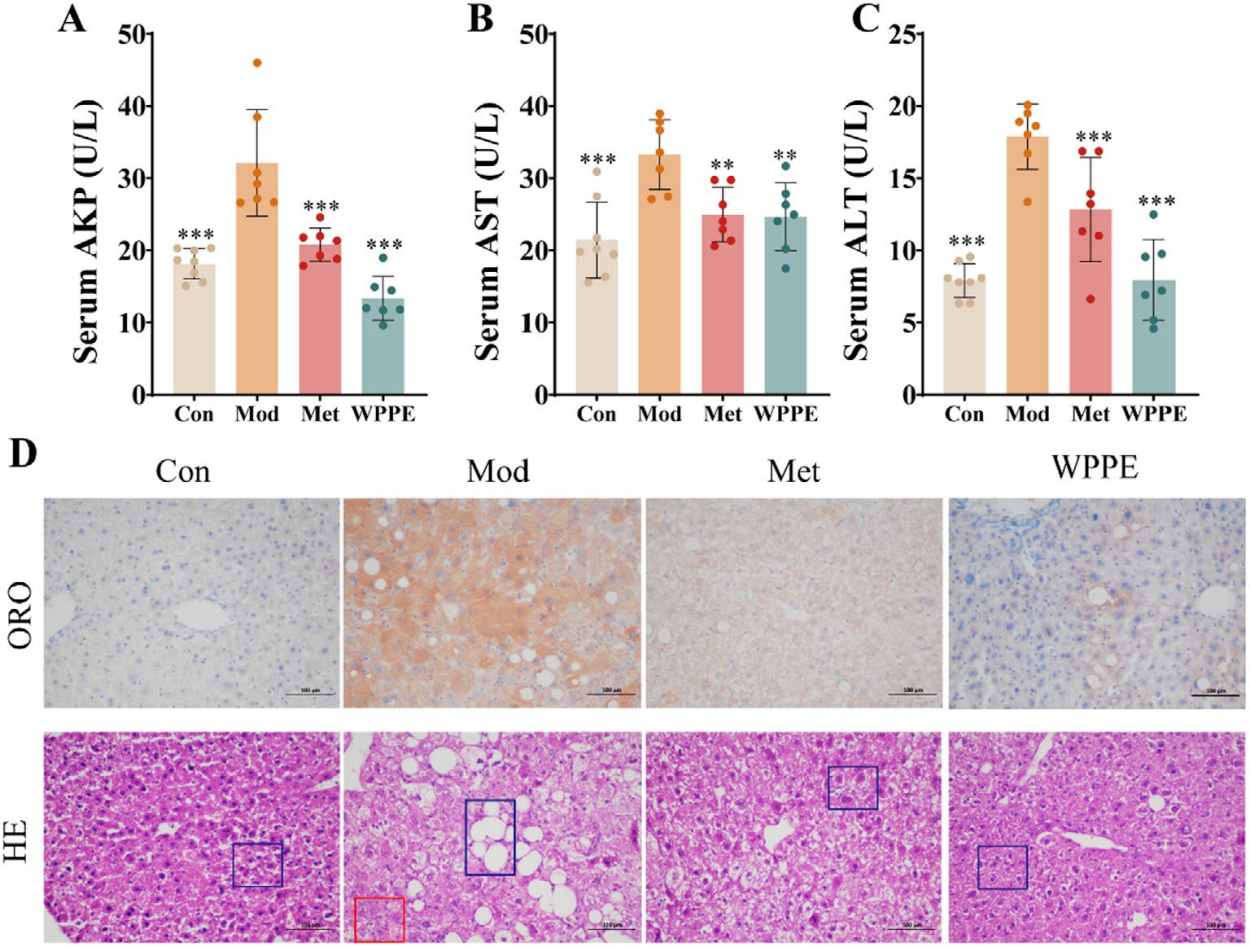
Effects of WPPE supplementation on (A) serum AKP, (B) serum AST, and (C) serum ALT levels, as well as (D) liver pathological tissue section (100×) in T2D mice. *p < 0.05, **p < 0.01, and ***p < 0.001 represented the significant difference of other groups with Mod group.

**FIGURE 3:**
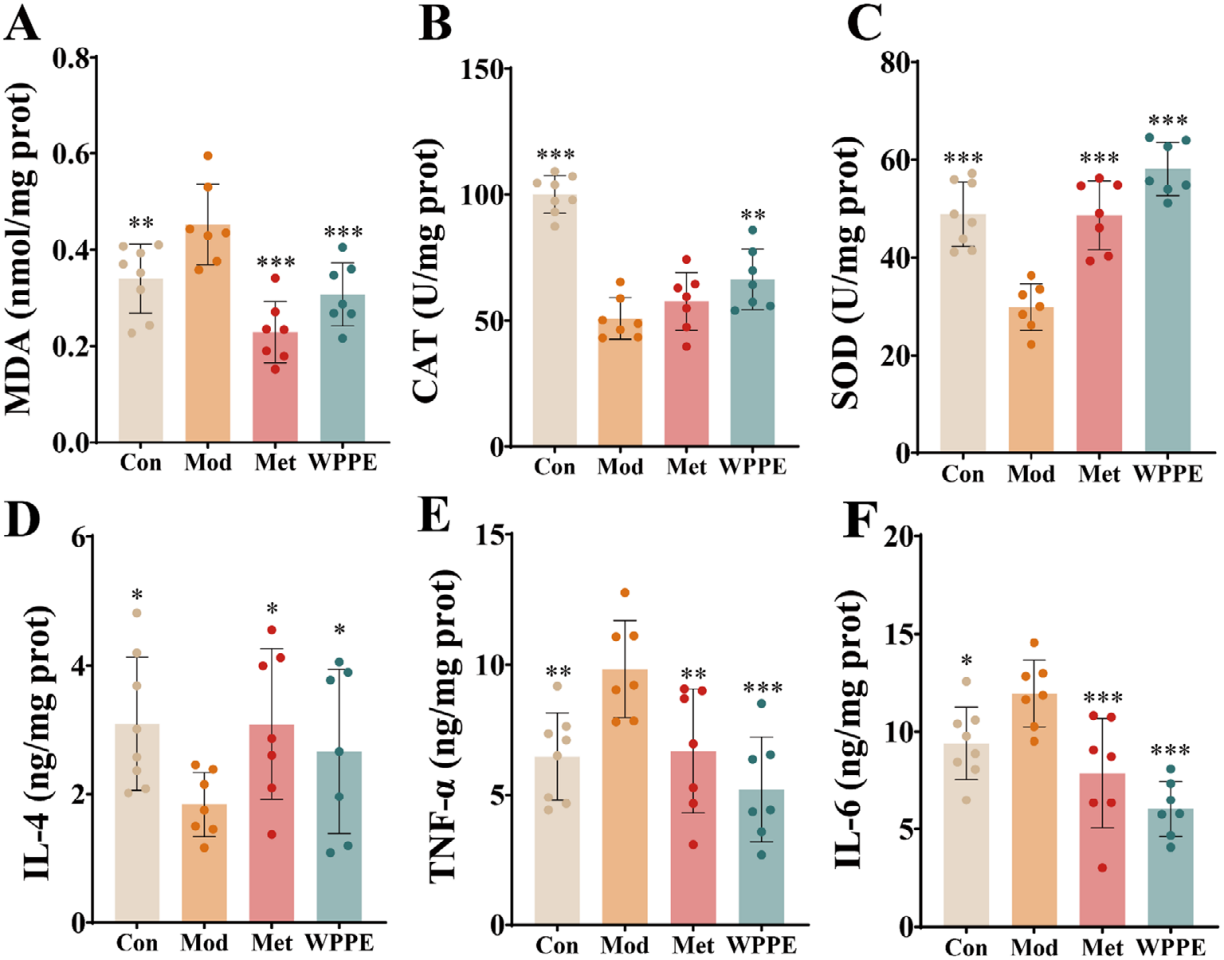
Effects of WPPE supplementation on hepatic oxidative stress and inflammation in T2D mice. (A) MDA content, (B) CAT level, (C) SOD level, (D) IL‐4 level, (E) TNF‐α level, and (F) IL‐6 level. *p < 0.05, **p < 0.01, and ***p < 0.001 represented the significant difference of other groups with Mod group.

AKP, ALT, and AST are important indicators of liver damage. According to Figure 2A–C, the levels of these enzymes were significantly elevated in the Mod group compared with the Con group, while they were recovered after WPPE treatment. The above results indicated that WPPE could alleviate T2DM‐associated liver injury. As shown in Figure 2D, the red and blue boxes respectively represented the lipid droplet and inflammatory cells. Histopathological examination demonstrated significant lipid vacuolization and inflammatory lesions in the hepatic tissue of T2DM mice, and the cell boundary was not obvious. WPPE treatment alleviated hepatic steatosis, including reducing lipid droplet accumulation as well as emptying the bright area and inflammatory cells. Furthermore, liver injury can accelerate oxidative stress and inflammation. Changes in the levels of CAT, SOD, MDA, and inflammatory cytokines in the liver were evaluated. It was also found that MDA content was increased (*p* < 0.05), while CAT and SOD activities were decreased in T2DM mice (Figure 3A–C). WPPE treatment attenuated hepatic oxidative stress by suppressing the imbalance of the above three key factors. Moreover, inflammation is highly related to the development of liver injury. T2DM induces the production of inflammatory factors. T2DM markedly elevated pro‐inflammatory cytokines (TNF‐α and IL‐6) and reduced anti‐inflammatory cytokines IL‐4 in mice. WPPE treatment decreased the TNF‐α and IL‐6 levels by 47.1% and 49.6%, respectively, while it increased the level of IL‐4 by 45.1% (*p* < 0.05) (Figure 3D–F). Taken together, WPPE may alleviate liver damage in T2DM mice by enhancing the production of antioxidant enzymes and attenuating inflammation.

### 3.3 Effect of WPPE on Target Genes of Liver Signaling Pathways

**FIGURE 4:**
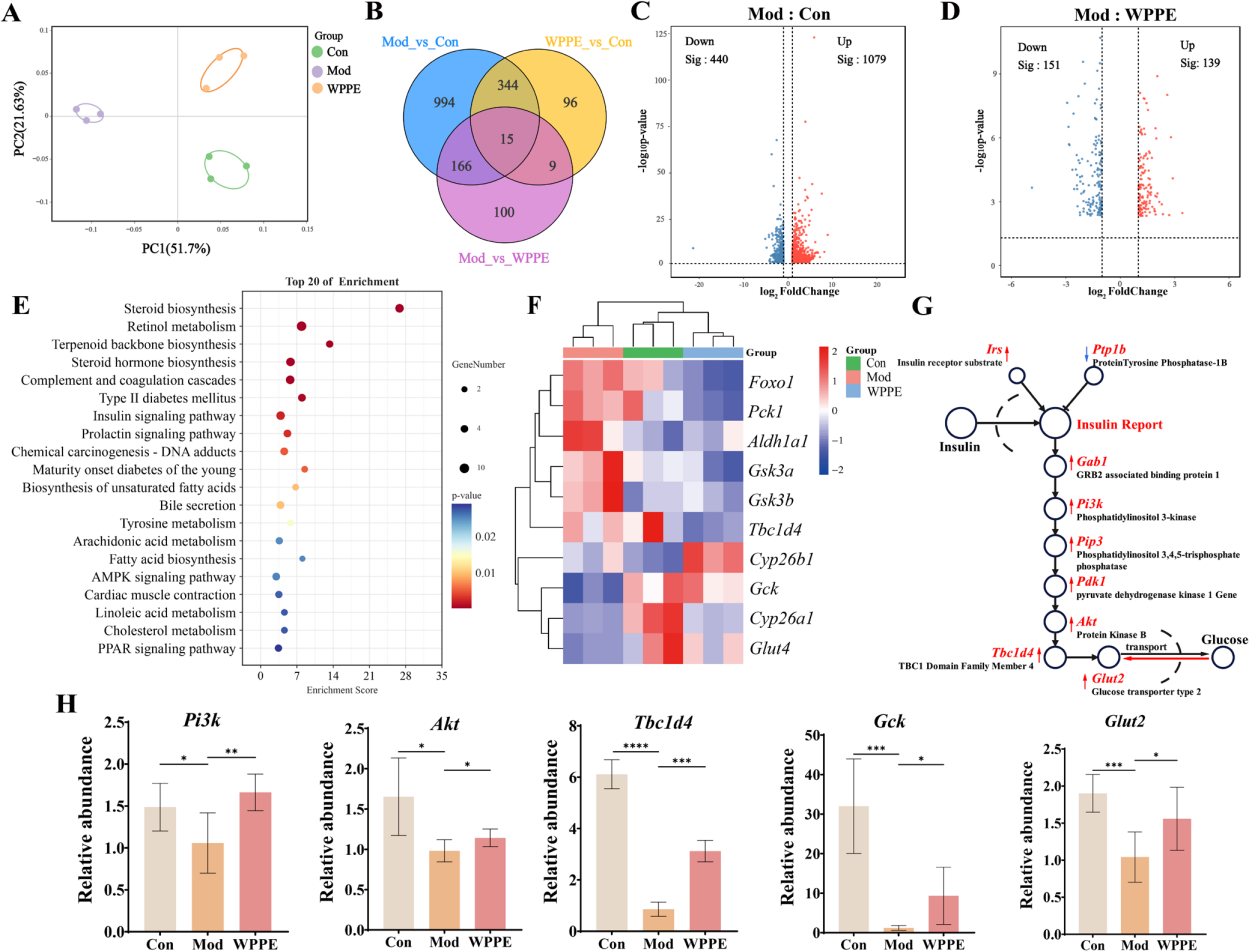
Hepatic transcriptome analysis revealed the molecular mechanism of WPPE regulating glucose metabolism in T2D mice. (A) PCA analysis based on hepatic transcriptomic data. (B) Venn diagram showing the DEGs among various groups. Volcano plots of DEGs in Mod versus Con (C) and Mod versus WPPE (D) groups. (E) Bubble plots exhibiting the principal enriched metabolic pathways of DEGs. (F) Heatmap of DEGs. (G) Schematic of the PI3K/AKT/TBC1D4 signaling pathway. (H) Validation of key gene expression in liver of mice. *p < 0.05, **p < 0.01, and ***p < 0.001 represented the significant difference of other groups with Mod group.

To investigate the molecular mechanisms underlying the regulatory effects of WPPE on glucose metabolic dysregulation in T2DM mice, hepatic transcriptomic analysis was performed (Figure 4). Principal coordinate analysis (PCA) result found that WPPE group exhibited a distinct separation with Mod group, while was near to control group, suggesting that WPPE treatment reversed the liver injury by modulating the key genes. As described in Figure 4B–D, a total of 290 DEGs was found in Mod versus WPPE groups; WPPE treatment upregulated 139 DEGs but downregulated 151 DEGs.

Additionally, the KEGG enrichment analysis result was listed in Figure 4E, the more red and bigger of the dots implied that the difference of these pathways between Mod and WPPE was more significant. The top pathways contained insulin signaling, steroid biosynthesis, retinol metabolism, terpenoid backbone biosynthesis, and were the key pathways regulated by WPPE in T2DM mice, which may be contributed to the improvement of basic liver indicators. Then, the target genes modulated by WPPE were further identified through hierarchical clustering analysis of gene expression profiles. Ten DEGs were subsequently confirmed via comparative analysis against the Mod group. Figure 4F demonstrates that WPPE up‐regulated the expression of key genes (*Cyp26b1*, *Cyp26a1*, *Gck*, *Glut4*), while downregulating the expression of *Foxo1*, *Pck1*, *Aldh1a1*, *Gsk3a*, *Gsk3b*, and *Tbc1d4*. These findings suggested that the identified genes played a crucial regulatory role in alleviating T2DM‐induced glucose metabolic dysregulation, particularly through modulating the insulin signaling pathway.

To verify the results of transcriptomic analysis, RT‐qPCR method was applied to assess the target genes. Additionally, the expression levels of key genes related to the insulin signaling pathway, such as *Pi3k*, *Akt*, *Tbc1d4*, and *Glut2*, were measured. As displayed in Figure 4H, WPPE treatment obviously suppressed the downregulation of hepatic *Pi3k*, *Akt*, *Tbc1d4*, and *Glut2* in T2DM mice (*p* < 0.05). Furthermore, the expression level of *Gck* was increased 679.1% in WPPE group by comparison with Mod group (*p* < 0.05). Thus, WPPE may ameliorate glucose metabolism disorders induced by T2DM through the PI3K/AKT/TBC1D4 pathway, which belongs to the insulin signaling pathway.

### 3.4 WPPE Alleviated Gut Microbiota Disorder in T2DM Mice

**FIGURE 5:**
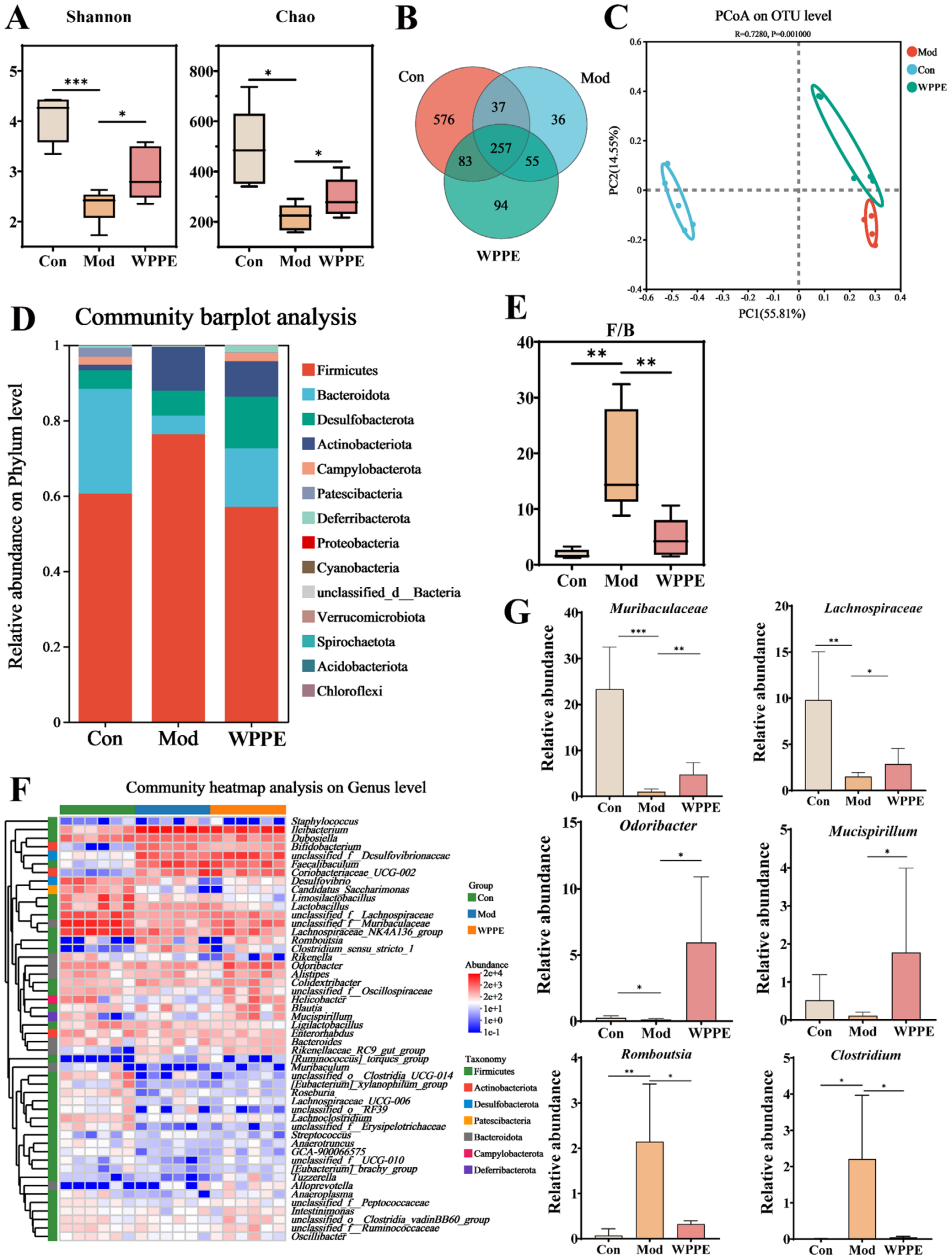
Effects of WPPE supplementation on gut microbiota composition in T2D mice. (A) Shannon and Chao indexes. (B) Venn diagram exhibiting similarities among different groups based on OUT. (C) PCoA of gut microbiota. (D) The relative abundance of gut microbiota in phylum level. (E) The ratio value of Firmicutes to Bacteroidetes (F/B). (F) Heatmap of commonly differentially gut microbiota in genus level. (G) The relative abundance of key gut microbiota. (*) *p* < 0.05, (**) *p* < 0.01 and (***) *p* < 0.001 represented the significant difference of other groups with Mod group.

Gut microbiota exhibited strong correlation with T2DM and its complications; therefore, the impact of WPPE on gut microbiota composition of T2DM mice was explored using 16S rRNA sequencing. Chao and Shannon indexes can reflect the change of alpha diversity in gut microbiota. According to Figure 5A, the T2DM mice supplemented with WPPE had higher species richness and diversity due to an increase of the Shannon index. Moreover, the Venn diagram exhibited 257 common genera in all groups (Figure 5B). The Con, Mod, and WPPE groups had 576, 36, and 94 exclusive genera, respectively. The beta diversity of gut microbiota was reflected by PCoA analysis on OUT level. The principal coordinate analysis (PCoA) result revealed a clear separation between the Con and Mod groups (Figure 5C), indicating that T2DM disrupted the balance of gut microbiota. However, WPPE treatment significantly reversed gut microbiota disorder in T2DM mice, and the microbiota composition was similar to that of the Con group. Community composition analysis found that the major microbiota at the phyla level were *Firmicutes*, *Bacteroidetes*, *Desulfobacterota*, and *Actinobacteria* (Figure 5D). WPPE treatment significantly increased the relative abundance of Bacteroidetes and reduced the F/B ratio (Figure 5E). At the genus level, compared with the Con group, the relative abundance of *Romboutsia* and *Clostridium* was increased, while the relative abundance of *Muribaculaceae*, *Lachnospiraceae*, *Odoribacter*, and *Mucispirillum* was decreased in the Mod group (Figure 5F). WPPE intervention successfully reversed these alterations caused by T2DM. Welch's *t*‐test was further conducted to distinguish the specific microbial biomarkers in three groups. WPPE treatment resulted in a significant increase in the relative abundance of *Muribaculaceae*, *Lachnospiraceae*, *Odoribacter*, and *Mucispirillum*, and was 0.90–45.58 folds of that in the Mod group (*p* < 0.05) (Figure 5G). These findings demonstrate that WPPE could modulate gut dysbiosis induced by T2DM through altering the relative abundance of beneficial and harmful bacteria.

### 3.5 WPPE Altered SCFAs Levels in T2DM Mice

**FIGURE 6:**
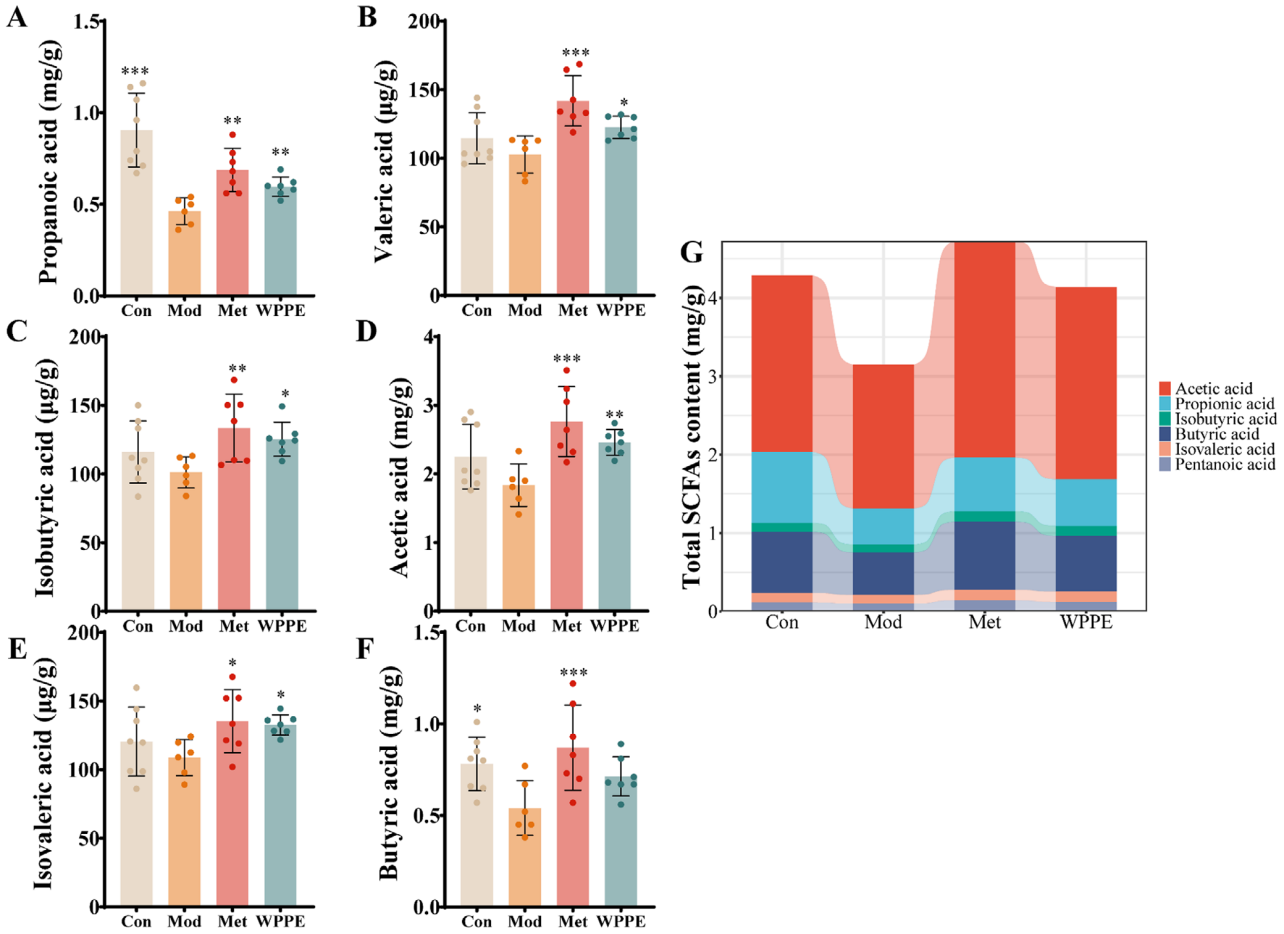
Effects of WPPE supplementation on the production of SCFAs in T2D mice. (A) Propionic acid. (B) Valeric acid. (C) Isobutyric acid. (D) Acetic acid. (E) Isovaleric acid. (F) Butyric acid. (G) Total SCFAs content. *p < 0.05, **p < 0.01, and ***p < 0.001 represented the significant difference of other groups with Mod group.

SCFAs, generated by the gut microbiota via fermenting indigestible carbohydrates, possess multifaceted biological activities, such as modulating energy expenditure, improving glucose metabolism, and displaying anti‐inflammatory and anticancer properties (Zhang et al. 2023). According to Figure 6, T2DM decreased the SCFAs levels in mice, while that in the WPPE group was comparable with normal mice. The levels of propionic acid, butyric acid, acetic acid, isobutyric acid, valproic acid, and isovaleric acid were respectively elevated by 30.4%, 31.5%, 33.9%, 23.9%, 19.3%, and 21.8% in the WPPE group than those in the Mod group (*p* < 0.05). The results suggest that WPPE can promote the production of SCFAs, which is in accord with the impact on gut microbiota composition.

### 3.6 Correlation Analysis Among Gut Microbiota, Key Genes, and Basic Indexes

**FIGURE 7:**
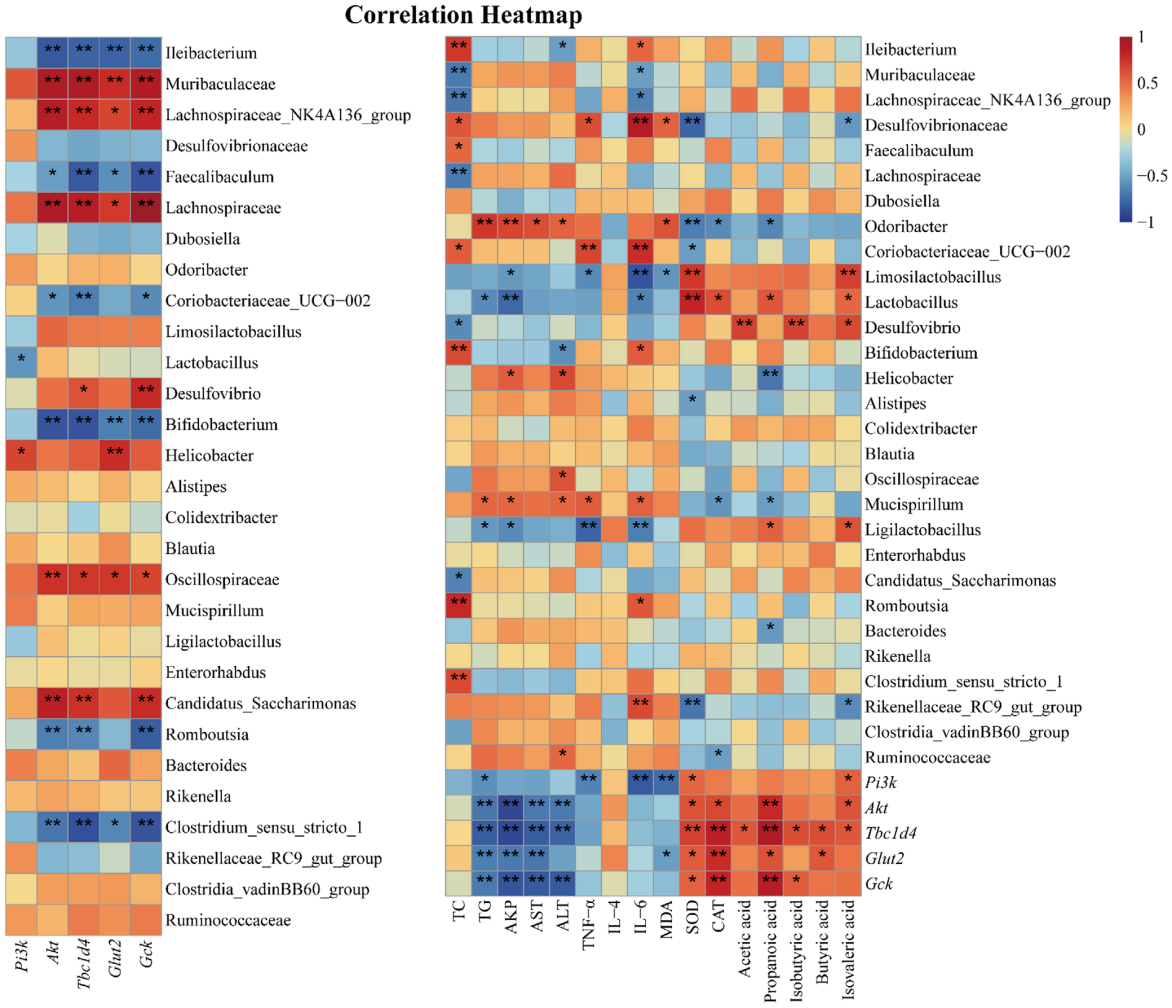
Spearman correlation analysis among biochemical parameters, top 29 gut microbiota and key genes relating with glucose metabolism. *p < 0.05; **p < 0.01; ***p < 0.001.

The relationships between biochemical parameters, gut microbiota, and key genes were explored using Spearman's correlation analysis and provided insights into their potential interplay and biological significance. As shown in Figure 7, the results indicated that most of the gut microbiota and key genes in the PI3K/AKT/TBC1D4 pathway were highly correlated with the biochemical parameters and liver indicators. Especially, *Tbc1d4*, *Gck*, and *Glut2* exhibited highly negative correlations with AKP, AST, valproic acid, and *Odoribacter*, while showing positive correlation with SOD and CAT. *Odoribacter* was closely related to MDA and SOD, but *Muribaculaceae*, *Mucispirillum*, and PI3K were negatively correlated with IL‐6. These findings indicated that WPPE may improve serum and liver parameters in T2DM mice through modulation of key gut microbiota and genes.

## 4 Discussion

In this study, the regulatory effect of WPPE on T2DM was investigated, including basic biochemical parameters, gut microbiota, and hepatic key genes related to glucose metabolism. Spearman's correlation analysis was applied to evaluate the interaction among these indexes. The results showed that WPPE intervention significantly lowered blood glucose, GSP, and HbA1c levels in T2DM mice, suggesting that WPPE had an excellent hypoglycemic effect. In addition, OGTT and ITT tests indicated that WPPE could improve glucose tolerance and IR by decreasing AUC values of T2DM mice. The HOMA‐IR and insulin levels also declined sharply after WPPE treatment. WPPE intervention remarkably enhanced the HDL‐C level, while reducing the TG, TC, and LDL‐C levels of T2DM mice. These findings proved that WPPE could maintain glucose and lipid homeostasis in T2DM mice. Varga et al. (2017) reported that *Prunus cerasus* seed extract treatment reduced the blood glucose level and relieved IR in diabetic rats, which is consistent with our results. WPPE showed good inhibitory capacity on carbohydrate enzymes like α‐glucosidase, which may contribute to the glucose‐lowering effect (Liu, Nisar, and Wan 2020). Our previous study found that the total phenolics of WPPE extract were 219.27 mg GAE/g E., quercetin, kaempferol, caffeoylquinic acid, and their derivatives were the major polyphenols in WPPE (Luo et al. 2024). Some, like apigenin and quercetin, showed good antidiabetic activity, and could effectively improve oral glucose tolerance, insulin resistance, and blood glucose levels in T2DM mice by modulating gut microbiota composition, SCFAs generation, and PI3K/AKT signal pathway (Miao et al. 2023; Ren et al. 2016; Yuan et al. 2024), which may explain our results.

The development of T2DM leads to varying degrees of liver damage (Sarić et al. 2009). WPPE treatment could improve liver injury by reversing the increase of AKP, ALT, and AST levels, which were also supported by histopathology observation. Moreover, T2DM is accompanied by the generation of excessive ROS and inflammatory cytokines and can exacerbate liver damage and IR (Rains and Jain 2011). SOD and CAT are primary antioxidant enzymes, and MDA is a marker of lipid peroxidation (Zhang et al. 2021a). WPPE intervention alleviated oxidative stress in T2DM mice by enhancing SOD and CAT levels as well as reducing MDA level. Liu, Chang, et al. (2020) discovered that *Cerasus humilis* polyphenol effectively alleviated oxidative stress by improving the ROS, SOD, and CAT levels, and the trend was similar to that in our study. WPPE has been reported to show strong antioxidant activity, and the identified phenolics like apigenin and quercetin‐3‐*O*‐glucoside were good antioxidants, which may contribute to the therapeutic effect (Li et al. 2016; Necip et al. 2024). In addition, inhibiting the production of IL‐6 and TNF‐α can modulate insulin sensitivity and pancreatic β‐cell damage, thereby lowering blood glucose (Hotamisligil 2017). An expansion of anti‐inflammatory factors IL‐4 and a reduction of pro‐inflammatory factors TNF‐α and IL‐6 were found after WPPE treatment, and the trend was in accordance with basic indicators involved in glucose metabolism. Cherry phenolics also could reduce the IL‐6 concentration in db/db mice, while the effect was weaker than that of WPPE (Noratto et al. 2018). These findings suggest that WPPE may relieve IR and liver damage by suppressing oxidative stress and inflammatory response.

The comprehensive hepatic transcriptome analysis revealed that WPPE treatment successfully reversed partly dysregulated hepatic transcriptomic profile and alleviated T2DM progression through the regulation of 10 key target genes. The effect of WPPE on *Gck*, *Tbc1d4*, and *Glut4* were more obvious than other genes, which were belong to the insulin signaling pathway. All these target genes were validated by RT‐qPCR analysis. Only the genes in insulin signaling pathway showed the significant differences, included *Pi3k*, *Akt*, *Tbc1d4*, *Glut2*, and *Gck*. PI3K/AKT pathway is belong to insulin signaling pathway, and contained the key upstream gene of *Akt* and *Pi3k*, which can maintain glucose homeostasis by reducing hepatic glucose production and enhancing glycogenesis storage (Titchenell et al. 2016). GLUT4 and GLUT2 are the key downstream genes of PI3K/AKT pathway, and *Glut2* can regulate glucose metabolism disorders by increasing glucose transport (Molinaro et al. 2019; Mueckler and Thorens 2013; Liu et al. 2022). *Tbc1d4* is direct target of *Akt*, and can facilitate the conversion of glucose to glucose‐6‐phosphate (G6P), then accelerate glycogen synthesis (Agius 2008). Additionally, many studies found that GLUT4 mainly existed in skeletal muscle and adipocytes, while the expression of it in liver was little and hard to detect (James et al. 1988; Thorens et al. 1988). WPPE could significantly up‐regulate the expression level of *Pi3k*, *Akt*, *Tbc1d4*, *Glut2*, and *Gck* in T2DM mice. Raspberry ketones treatment reduced blood glucose levels by rising the gene expression of *Gck*, which was consistent with our study (Zhu, Zhang, et al. 2024). Mohamed et al. (2024) also observed that *Coccoloba uvifera* leaves polyphenols extract alleviated IR by modulating *Irs1*/*Pi3k*/*Akt*/*Glut2* signaling pathways. The identified phenolics like rutin (Liang et al. 2018) and gallic acid (Variya et al. 2020) in WPPE also have been demonstrated to relieve IR through PI3K/AKT signaling pathway. Zhou et al. (2023) and Chike‐Ekwughe et al. (2024) also found that phenolics extract could regulate hepatic glucose metabolism disorders of T2DM through PI3K/AKT pathway. Spearman's correlation analysis indicated that *Gck*, *Glut2*, and *Tbc1d4* were closely related with liver function and oxidative stress indexes like CAT, SOD, and AST. In summary, WPPE may ameliorate glucose metabolism disorder through regulating the target genes of PI3K/AKT/TBC1D4 pathways in T2DM mice, especially for an increment in glucose uptake and hepatic gluconeogenesis.

**FIGURE 8:**
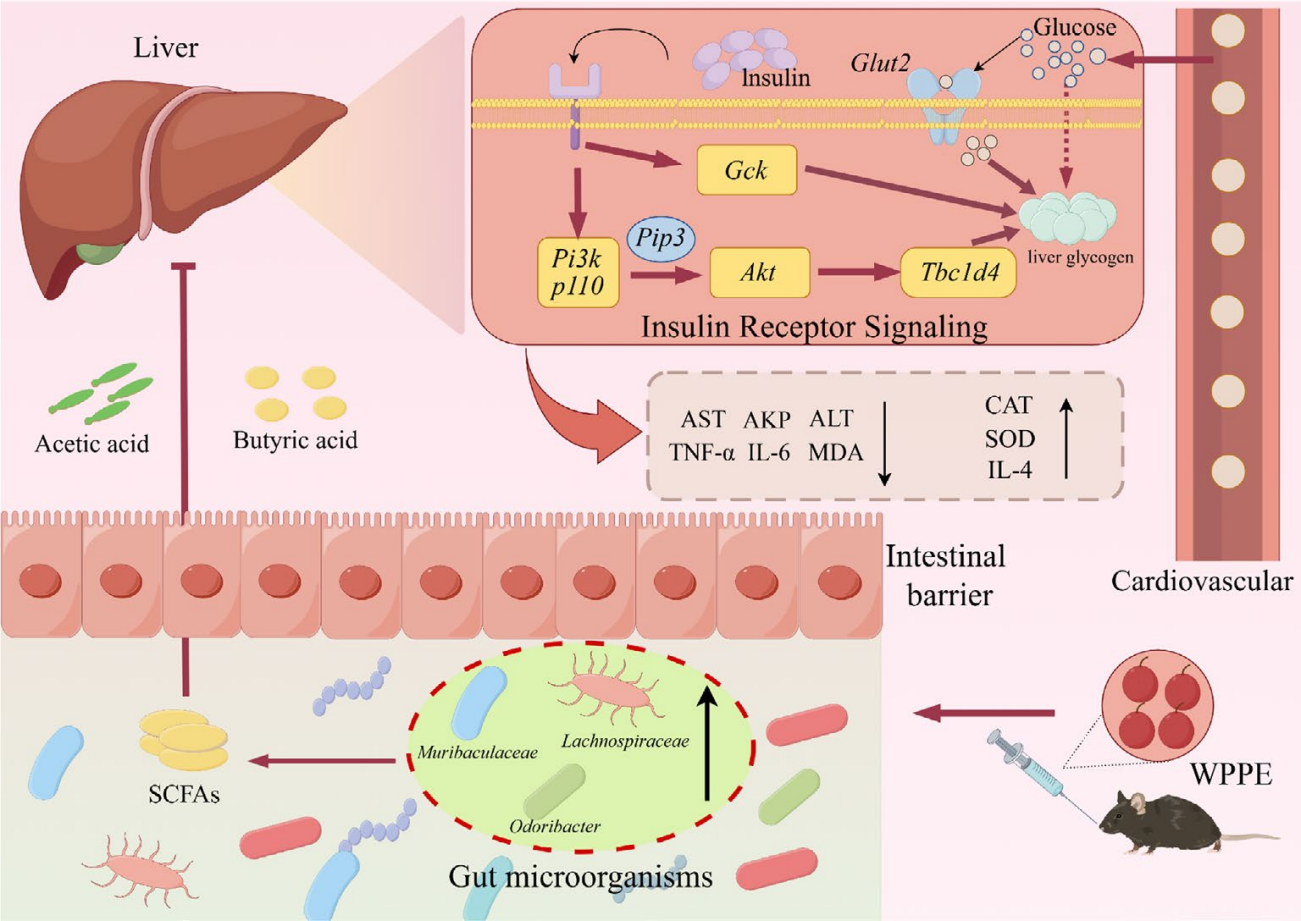
Schematic diagram exhibiting the effects of WPPE on T2D and the hypoglycemic mechanisms.

Many studies proved that gut microbiota and its metabolites can influence host glucose metabolism, and show strong relation with metabolic diseases, which is a potential therapeutic target for T2DM (Wang et al. 2022). WPPE treatment changed the diversity and composition of gut microbiota, and characterized by a rising of Shannon index and clear separation in PCA analysis, indicating WPPE reversed gut microbiota disorder induced by T2DM. F/B is a potential biomarker for gut health, and is highly related with gut inflammation (Wen et al. 2023). The relative abundance of Bacteroidota was increased, while Firmicutes/Bacteroidota (F/B) ratio was reduced after WPPE supplementation. *Muribaculaceae*, a genus belongs to Bacteroidota, can promote the production of butyric acid and improve glucose metabolism and IR (Zhu, Chen, et al. 2024). *Odoribacter* is also a butyrate‐producing bacterium to be able to alleviate glucose tolerance and inflammatory in db/db mice (Huber‐Ruano et al. 2022). *Lachnospiraceae* has been demonstrated to regulate T2DM by affecting carbohydrates and energy metabolism (Vacca et al. 2020). *Mucispirillum* can use SCFAs for energy metabolism, and improve oxidative stress and inflammatory (Loy et al. 2017; Zhang et al. 2021b). *Romboustia* is highly associated with liver function, and *Clostridum* was found to show high content in T2DM patients (Li and Bu 2020). WPPE treatment significantly enhanced the relative abundance of *Muribaculaceae*, *Lachnospiraceae*, *Odoribacter*, and *Mucispirillum*, while decreased relative abundance of *Romboutsia* and *Clostridium*. These findings was consistent with the report of Li et al. (2021). In addition, *Muribaculaceae* and *Odoribacter* were highly correlated with hepatic basic indicators like IL‐6 and SOD. These gut microbiota may be the key target for preventing T2DM. Microbiota‐derived SCFAs are vital in modulating glycometabolism, and can improve blood glucose level, IR, and hepatic damage (Puddu et al. 2014). This study showed that WPPE treatment significant increased fecal butyric, propionic and valeric acids levels in T2DM mice. Yan et al. (2025) also observed that pumpkin components obviously increased fecal SCFA levels in db/db mice, and then inhibited gluconeogenesis and IR, resulting in reduced fasting blood glucose. Propionic acid can enhance glucose tolerance, suppress hepatic gluconeogenesis and regulate *Pi3k*/*Akt* signaling pathway (Wu et al. 2022; Zadeh‐Tahmasebi et al. 2016). Butyric acid has been observed to prevent and treat IR, and can promote carbohydrate consumption (Gao et al. 2009). Valeric acid can enhance glucose uptake (Han et al. 2014). Butyric and propionic acids display positive relationship with *Glut2* and *Tbc1d4*. And many studies also demonstrated that SCFAs could promote the expression of MCT1 and GPR43 in liver of T2DM mice, and then affected the PI3K/AKT signal pathway (Chatterjee et al. 2016; Chen et al. 2024; Zhang et al. 2019). Therefore, WPPE supplementation could promote the production of beneficial bacteria like *Muribaculaceae* and SCFAs like butyric acid. While, SCFAs can further enter in liver, and may relieve glucose metabolism disorder of T2DM mice by activating the key gene in insulin signaling pathway, leading to the alteration in blood glucose level, inflammation and IR of T2DM mice. As displayed in Figure 8, the schematic diagram illustrated the underlying molecular mechanisms of WPPE in ameliorating hyperglycemia of T2DM mice.

## 5 Conclusion

In summary, this study revealed the hypoglycemic effect of WPPE in T2DM mice and its underlying mechanisms. WPPE treatment decreased the blood glucose level, improved glucose tolerance and IR in T2DM mice. Moreover, WPPE treatment enhanced antioxidant enzyme activities and lowered inflammation, thereby relieving liver damage in T2DM mice. Further, hepatic transcriptome analysis indicated that WPPE supplementation could regulate glucose metabolism disorders by modulating the key gene expressions in the PI3K/AKT/TBC1D4 pathway, including *Pi3k*, *Akt*, *Glut2*, *Tbc1d4*, and *Gck*. WPPE supplementation restored gut microbiota dysbiosis induced by T2DM, leading to the increased abundance in *Muribaculaceae*, *Lachnospiraceae*, *Odoribacter*, and *Mucispirillum*, as well as the decreased abundance in *Romboutsia* and *Clostridium*. The production of SCFAs, especially propionic and butyric acids, was significantly increased in T2DM mice after WPPE treatment. Overall, a significant correlation among the modulation of inflammation, oxidative stress, gut microbiota, and the PI3K/AKT/TBC1D4 pathway was observed underlying the hypoglycemic effect of WPPE. In the future work, fecal transplantation and gene knock‐out experiments need to be carried out to verify the key roles of gut microbiota and hepatic genes modulated by WPPE on alleviating diabetes mellitus. The specific individual phenolics with good hypoglycemic activity should also be further isolated from WPPE.

## Author Contributions

**Xinpeng Cheng:** investigation, validation, formal analysis, writing – original draft. **Xing Xie:** methodology, supervision, investigation, writing – review and editing. **Shibo Luo:** methodology, data curation. **Quanyuan Xie:** software, formal analysis, writing – review and editing. **Haiyan Xiang:** investigation, software, formal analysis. **Chunyan Peng:** investigation, software, formal analysis. **Qiao Ding:** supervision. **Hongbing Fan:** methodology, writing – review and editing. **Wei Liu:** conceptualization, supervision, funding acquisition. **Lu Zhang:** conceptualization, supervision, formal analysis, writing – review and editing, data curation, funding acquisition.

## Funding

This research was supported by the Natural Science Foundation of Xinjiang Uygur Autonomous Region (2022D01C455), Natural Science Foundation of Jiangxi Province (20232BAB215063), and Department of Education of Jiangxi Province (GJJ2200382).

## Ethics Statement

C57BL/6J mice were purchased from Charles River Laboratories (Beijing, China). All animal procedures were performed in accordance with the Guidelines for Care and Use of Laboratory Animals of Nanchang Research Institute in Sun Yat‐sen University and approved by the Animal Ethics Committee of Nanchang Research Institute in Sun Yat‐sen University (number of permit: SYSUNC‐IACUC‐B‐2025‐0001).

## Conflicts of Interest

The authors declare no conflicts of interest.

## Supporting information


**Appendix S1:** fsn371498‐sup‐0001‐AppendixS1.docx.

## Data Availability

The data that support the findings of this study are available from the corresponding author.

## References

[fsn371498-bib-0001] Agius, L. 2008. “Glucokinase and Molecular Aspects of Liver Glycogen Metabolism.” Biochemical Journal 414, no. 1: 1–18. 10.1042/BJ20080595. 18651836

[fsn371498-bib-0002] Bauer, K. C. , P. T. Littlejohn , V. Ayala , A. Creus‐Cuadros , and B. B. Finlay . 2022. “Nonalcoholic Fatty Liver Disease and the Gut‐Liver Axis: Exploring an Undernutrition Perspective.” Gastroenterology 162, no. 7: 1858–1875.e2. 10.1053/j.gastro.2022.01.058. 35248539

[fsn371498-bib-0003] Blandino, G. , R. Inturri , F. Lazzara , M. Di Rosa , and L. Malaguarnera . 2016. “Impact of Gut Microbiota on Diabetes Mellitus.” Diabetes & Metabolism 42, no. 5: 303–315. 10.1016/j.diabet.2016.04.004. 27179626

[fsn371498-bib-0004] Cevallos‐Casals, B. A. , D. Byrne , W. R. Okie , and L. Cisneros‐Zevallos . 2006. “Selecting New Peach and Plum Genotypes Rich in Phenolic Compounds and Enhanced Functional Properties.” Food Chemistry 96, no. 2: 273–280. 10.1016/j.foodchem.2005.02.032.

[fsn371498-bib-0005] Chatterjee, I. , A. Kumar , A. N. Anbazhagan , W. A. Alrefai , A. Borthakur , and P. K. Dudeja . 2016. “Butyrate Enhances MCT1 Association With CD147 via GPR109A Activation‐Dependent Mechanisms.” FASEB Journal 30, no. S1: 1020.3. 10.1096/fasebj.30.1_supplement.1020.3.

[fsn371498-bib-0006] Chen, M. , P. Pan , H. Zhang , R. Li , D. Ren , and B. Jiang . 2024. “ *Latilactobacillus sakei* QC9 Alleviates Hyperglycaemia in High‐Fat Diet and Streptozotocin‐Induced Type 2 Diabetes Mellitus Mice via the Microbiota‐Gut‐Liver Axis.” Food & Function 15, no. 15: 8008–8029. 10.1039/d4fo02316a. 38984868

[fsn371498-bib-0007] Chike‐Ekwughe, A. , L. B. John‐Africa , A. H. Adebayo , and O. O. Ogunlana . 2024. “Antioxidative and Anti‐Diabetic Effects of *Tapinanthus cordifolius* Leaf Extract on High‐Fat Diet and Streptozotocin‐Induced Type 2 Diabetic Rats.” Biomedicine & Pharmacotherapy 176: 116774. 10.1016/j.biopha.2024.116774. 38820976

[fsn371498-bib-0008] Colagiuri, S. , and A. Ceriello . 2025. Idf Diabetes Atlas. 11th ed. International Diabetes Federation. https://diabetesatlas.org/resources/idf‐diabetes‐atlas‐2025/.

[fsn371498-bib-0009] Cole, J. B. , and J. C. Florez . 2020. “Genetics of Diabetes Mellitus and Diabetes Complications.” Nature Reviews Nephrology 16, no. 7: 377–390. 10.1038/s41581-020-0278-5. 32398868 PMC9639302

[fsn371498-bib-0010] Dahlén, A. D. , G. Dashi , I. Maslov , et al. 2021. “Trends in Antidiabetic Drug Discovery: FDA Approved Drugs, New Drugs in Clinical Trials and Global Sales.” Frontiers in Pharmacology 12: 807548. 10.3389/fphar.2021.807548. 35126141 PMC8807560

[fsn371498-bib-0011] Deng, N. , Z. He , R. Guo , B. Zheng , T. Li , and R. H. Liu . 2020. “Highland Barley Whole Grain ( *Hordeum vulgare* L.) Ameliorates Hyperlipidemia by Modulating Cecal Microbiota, miRNAs, and AMPK Pathways in Leptin Receptor‐Deficient db/db Mice.” Journal of Agricultural and Food Chemistry 68, no. 42: 11735–11746. 10.1021/acs.jafc.0c04780. 32985184

[fsn371498-bib-0012] Gao, Z. , J. Yin , J. Zhang , et al. 2009. “Butyrate Improves Insulin Sensitivity and Increases Energy Expenditure in Mice.” Diabetes 58, no. 7: 1509–1517. 10.2337/db08-1637. 19366864 PMC2699871

[fsn371498-bib-0013] Gündüz, K. , and O. Saraçoğlu . 2012. “Variation in Total Phenolic Content and Antioxidant Activity of *Prunus cerasifera* Ehrh. Selections From Mediterranean Region of Turkey.” Scientia Horticulturae 134: 88–92. 10.1016/j.scienta.2011.11.003.

[fsn371498-bib-0014] Han, J. H. , I. S. Kim , S. H. Jung , S. G. Lee , H. Y. Son , and C. S. Myung . 2014. “The Effects of Propionate and Valerate on Insulin Responsiveness for Glucose Uptake in 3T3‐L1 Adipocytes and C2C12 Myotubes via G Protein‐Coupled Receptor 41.” PLoS One 9, no. 4: e95268. 10.1371/journal.pone.0095268. 24748202 PMC3991595

[fsn371498-bib-0015] Hotamisligil, G. S. 2017. “Inflammation, Metaflammation and Immunometabolic Disorders.” Nature 542, no. 7640: 177–185. 10.1038/nature21363. 28179656

[fsn371498-bib-0017] Huber‐Ruano, I. , E. Calvo , J. Mayneris‐Perxachs , et al. 2022. “Orally Administered *Odoribacter laneus* Improves Glucose Control and Inflammatory Profile in Obese Mice by Depleting Circulating Succinate.” Microbiome 10, no. 1: 135. 10.1186/s40168-022-01306-y. 36002880 PMC9404562

[fsn371498-bib-0018] James, D. E. , R. Brown , J. Navarro , and P. F. Pilch . 1988. “Insulin‐regulatable Tissues Express a Unique Insulin‐Sensitive Glucose Transport Protein.” Nature 333, no. 6169: 183–185. 10.1038/333183a0. 3285221

[fsn371498-bib-0019] Jiang, S. , A. Liu , W. Ma , et al. 2023. “ *Lactobacillus gasseri* CKCC1913 Mediated Modulation of the Gut‐Liver Axis Alleviated Insulin Resistance and Liver Damage Induced by Type 2 Diabetes.” Food & Function 14, no. 18: 8504–8520. 10.1039/d3fo01701j. 37655696

[fsn371498-bib-0020] Li, R. , and S. Bu . 2020. “Effects of Metformin on the Intestinal Microbiota of Rats With High‐Fat Diet‐Induced Non‐Alcoholic Fatty Liver Disease‐All Databases.” Chinese Journal of Digestion 40, no. 12: 861–867. 10.3760/cma.j.cn311367-20200506-00294.

[fsn371498-bib-0021] Li, X. , Q. Jiang , T. Wang , J. Liu , and D. Chen . 2016. “Comparison of the Antioxidant Effects of Quercitrin and Isoquercitrin: Understanding the Role of the 6″‐OH Group.” Molecules 21, no. 9: 1246. 10.3390/molecules21091246. 27657022 PMC6273918

[fsn371498-bib-0022] Li, Z. R. , R. B. Jia , and J. Wu . 2021. “Sargassum Fusiforme Polysaccharide Partly Replaces Acarbose Against Type 2 Diabetes in Rats.” International Journal of Biological Macromolecules 170: 447–458. 10.1016/j.ijbiomac.2020.12.126. 33352159

[fsn371498-bib-0023] Liang, W. , D. Zhang , J. Kang , et al. 2018. “Protective Effects of Rutin on Liver Injury in Type 2 Diabetic db/db Mice.” Biomedicine & Pharmacotherapy 107: 721–728. 10.1016/j.biopha.2018.08.046. 30138894

[fsn371498-bib-0024] Liu, P. , L. Jiang , W. Kong , et al. 2022. “PXR Activation Impairs Hepatic Glucose Metabolism Partly *via* Inhibiting the HNF4*α*–GLUT2 Pathway.” Acta Pharmaceutica Sinica B 12, no. 5: 2391–2405. 10.1016/j.apsb.2021.09.031. 35646519 PMC9136535

[fsn371498-bib-0025] Liu, S. , X. Chang , J. Yu , and W. Xu . 2020. “ *Cerasus humilis* Cherry Polyphenol Reduces High‐Fat Diet‐Induced Obesity in C57BL/6 Mice by Mitigating Fat Deposition, Inflammation, and Oxidation.” Journal of Agricultural and Food Chemistry 68, no. 15: 4424–4436. 10.1021/acs.jafc.0c01617. 32227855

[fsn371498-bib-0026] Liu, W. , M. Nisar , and C. Wan . 2020. “Characterization of Phenolic Constituents From *Prunus cerasifera* Ldb Leaves.” Journal of Chemistry 2020, no. 1: 1–5. 10.1155/2020/5976090.

[fsn371498-bib-0027] Loy, A. , C. Pfann , M. Steinberger , et al. 2017. “Lifestyle and Horizontal Gene Transfer‐Mediated Evolution of *Mucispirillum schaedleri*, a Core Member of the Murine Gut Microbiota.” mSystems 2, no. 1: e00171‐16. 10.1128/mSystems.00171-16. 28168224 PMC5285517

[fsn371498-bib-0028] Luo, S. , W. Liu , X. Xie , et al. 2024. “Antioxidant and Hypoglycemic Activities of Purple *Prunus cerasifera* Ehrh Extracts and Identification of Chemical Constituents.” Food and Fermentation Industries 50, no. 19: 191–200. 10.13995/j.cnki.11-1802/ts.039997.

[fsn371498-bib-0029] Miao, L. , H. Zhang , M. S. Cheong , et al. 2023. “Anti‐Diabetic Potential of Apigenin, Luteolin, and Baicalein via Partially Activating PI3K/Akt/Glut‐4 Signaling Pathways in Insulin‐Resistant HepG2 Cells.” Food Science and Human Wellness 12, no. 6: 1991–2000. 10.1016/j.fshw.2023.03.021.

[fsn371498-bib-0030] Mohamed, F. A. , R. H. Sayed , M. N. A. Khalil , M. A. Salem , A. S. El Senousy , and A. M. El‐Halawany . 2024. “Ameliorative Activity of Standardized *Coccoloba uvifera* Leaves Extract Against Streptozotocin‐Induced Diabetic Rats via Activation of IRS‐1/PI3K/AKT/GLUT2 Pathway in Liver.” Future Journal of Pharmaceutical Sciences 10, no. 1: 132. 10.1186/s43094-024-00707-0.

[fsn371498-bib-0031] Molinaro, A. , B. Becattini , A. Mazzoli , et al. 2019. “Insulin‐Driven PI3K‐AKT Signaling in the Hepatocyte Is Mediated by Redundant PI3Kα and PI3Kβ Activities and Is Promoted by RAS.” Cell Metabolism 29, no. 6: 1400–1409.e5. 10.1016/j.cmet.2019.03.010. 30982732

[fsn371498-bib-0032] Moruzzi, M. , N. Klöting , M. Blüher , et al. 2021. “Tart Cherry Juice and Seeds Affect Pro‐Inflammatory Markers in Visceral Adipose Tissue of High‐Fat Diet Obese Rats.” Molecules (Basel, Switzerland) 26, no. 5: 1403. 10.3390/molecules26051403. 33807712 PMC7961347

[fsn371498-bib-0033] Mueckler, M. , and B. Thorens . 2013. “The SLC2 (GLUT) Family of Membrane Transporters.” Molecular Aspects of Medicine 34, no. 2: 121–138. 10.1016/j.mam.2012.07.001. 23506862 PMC4104978

[fsn371498-bib-0034] Necip, A. , I. Demirtas , S. E. Tayhan , et al. 2024. “Isolation of Phenolic Compounds From Eco‐Friendly White Bee Propolis: Antioxidant, Wound‐Healing, and Anti‐Alzheimer Effects.” Food Science & Nutrition 12, no. 3: 1928–1939. 10.1002/fsn3.3888. 38455224 PMC10916560

[fsn371498-bib-0035] Noratto, G. D. , N. N. Lage , B. P. Chew , S. U. Mertens‐Talcott , S. T. Talcott , and M. L. Pedrosa . 2018. “Non‐Anthocyanin Phenolics in Cherry ( *Prunus avium* l.) Modulate IL‐6, Liver Lipids and Expression of PPARδ and LXRs in Obese Diabetic (db/db) Mice.” Food Chemistry 266: 405–414. 10.1016/j.foodchem.2018.06.020. 30381205

[fsn371498-bib-0036] Priyadarshini, M. , B. Wicksteed , G. E. Schiltz , A. Gilchrist , and B. T. Layden . 2016. “SCFA Receptors in Pancreatic β Cells: Novel Diabetes Targets?” Trends in Endocrinology and Metabolism: TEM 27, no. 9: 653–664. 10.1016/j.tem.2016.03.011. 27091493 PMC4992600

[fsn371498-bib-0037] Puddu, A. , R. Sanguineti , F. Montecucco , and G. L. Viviani . 2014. “Evidence for the Gut Microbiota Short‐Chain Fatty Acids as Key Pathophysiological Molecules Improving Diabetes.” Mediators of Inflammation 2014: 162021. 10.1155/2014/162021. 25214711 PMC4151858

[fsn371498-bib-0038] Rains, J. L. , and S. K. Jain . 2011. “Oxidative Stress, Insulin Signaling, and Diabetes.” Free Radical Biology & Medicine 50, no. 5: 567–575. 10.1016/j.freeradbiomed.2010.12.006. 21163346 PMC3557825

[fsn371498-bib-0039] Ren, B. , W. Qin , F. Wu , et al. 2016. “Apigenin and Naringenin Regulate Glucose and Lipid Metabolism, and Ameliorate Vascular Dysfunction in Type 2 Diabetic Rats.” European Journal of Pharmacology 773: 13–23. 10.1016/j.ejphar.2016.01.002. 26801071

[fsn371498-bib-0040] Saraswathi, K. , C. Sivaraj , and P. Arumugam . 2020. “Antioxidant and Antibacterial Activities of Ethanol Fruit Extract of Cherry Plum—*Prunus cerasifera* Ehrh.” Journal of Drug Delivery and Therapeutics 10, no. 1‐s: 45–50. 10.22270/jddt.v10i1-s.3851.

[fsn371498-bib-0041] Sarić, A. , S. Sobocanec , T. Balog , et al. 2009. “Improved Antioxidant and Anti‐Inflammatory Potential in Mice Consuming Sour Cherry Juice (* Prunus cerasus cv*. Maraska).” Plant Foods for Human Nutrition 64, no. 4: 231–237. 10.1007/s11130-009-0135-y. 19763832

[fsn371498-bib-0042] Thorens, B. , H. K. Sarkar , H. R. Kaback , and H. F. Lodish . 1988. “Cloning and Functional Expression in Bacteria of A Novel Glucose Transporter Present in Liver, Intestine, Kidney, and β‐Pancreatic Islet Cells.” Cell 55, no. 2: 281–290. 10.1016/0092-8674(88)90051-7. 3048704

[fsn371498-bib-0043] Titchenell, P. M. , W. J. Quinn , M. Lu , et al. 2016. “Direct Hepatocyte Insulin Signaling Is Required for Lipogenesis but Is Dispensable for the Suppression of Glucose Production.” Cell Metabolism 23, no. 6: 1154–1166. 10.1016/j.cmet.2016.04.022. 27238637 PMC4909537

[fsn371498-bib-0044] Vacca, M. , G. Celano , F. M. Calabrese , P. Portincasa , M. Gobbetti , and M. de Angelis . 2020. “The Controversial Role of Human Gut *Lachnospiraceae* .” Microorganisms 8, no. 4: 573. 10.3390/microorganisms8040573. 32326636 PMC7232163

[fsn371498-bib-0045] Varga, B. , D. Priksz , N. Lampé , et al. 2017. “Protective Effect of *Prunus cerasus* (Sour Cherry) Seed Extract on the Recovery of Ischemia/Reperfusion‐Induced Retinal Damage in Zucker Diabetic Fatty Rat.” Molecules (Basel, Switzerland) 22, no. 10: 1782. 10.3390/molecules22101782. 29065463 PMC6151469

[fsn371498-bib-0046] Variya, B. C. , A. K. Bakrania , and S. S. Patel . 2020. “Antidiabetic Potential of Gallic Acid From *Emblica officinalis* : Improved Glucose Transporters and Insulin Sensitivity Through PPAR‐γ and Akt Signaling.” Phytomedicine: International Journal of Phytotherapy and Phytopharmacology 73: 152906. 10.1016/j.phymed.2019.152906. 31064680

[fsn371498-bib-0047] Wang, G. , J. Song , Y. Huang , et al. 2022. “ *Lactobacillus plantarum* SHY130 Isolated From Yak Yogurt Attenuates Hyperglycemia in C57BL/6J Mice by Regulating the Enteroinsular Axis.” Food & Function 13, no. 2: 675–687. 10.1039/D1FO02387J. 34935020

[fsn371498-bib-0048] Wang, Y. , X. Chen , Y. Zhang , and X. Chen . 2012. “Antioxidant Activities and Major Anthocyanins of Myrobalan Plum ( *Prunus cerasifera* Ehrh.).” Journal of Food Science 77, no. 4: C388–C393. 10.1111/j.1750-3841.2012.02624.x. 22432436

[fsn371498-bib-0049] Wen, J. J. , M. Z. Li , C. H. Chen , et al. 2023. “Tea Polyphenol and Epigallocatechin Gallate Ameliorate Hyperlipidemia via Regulating Liver Metabolism and Remodeling Gut Microbiota.” Food Chemistry 404, no. pt. A: 134591. 10.1016/j.foodchem.2022.134591. 36444016

[fsn371498-bib-0050] Wu, Q. , J. Dong , X. Bai , et al. 2022. “Propionate Ameliorates Diabetes‐Induced Neurological Dysfunction Through Regulating the PI3K/Akt/eNOS Signaling Pathway.” European Journal of Pharmacology 925: 174974. 10.1016/j.ejphar.2022.174974. 35490725

[fsn371498-bib-0051] Yan, F. , X. Wang , and Y. Du . 2025. “Pumpkin Soluble Dietary Fiber Instead of Insoluble One Ameliorates Hyperglycemia via the Gut Microbiota‐Gut‐Liver Axis in db/db Mice.” Journal of Agricultural and Food Chemistry 73, no. 2: 1293–1307. 10.1021/acs.jafc.4c08986. 39811930

[fsn371498-bib-0052] Yuan, M. , T. Sun , Y. Zhang , et al. 2024. “Quercetin Alleviates Insulin Resistance and Repairs Intestinal Barrier in db/db Mice by Modulating Gut Microbiota.” Nutrients 16, no. 12: 1870. 10.3390/nu16121870. 38931226 PMC11206920

[fsn371498-bib-0053] Zadeh‐Tahmasebi, M. , F. A. Duca , B. A. Rasmussen , et al. 2016. “Activation of Short and Long Chain Fatty Acid Sensing Machinery in the Ileum Lowers Glucose Production In Vivo.” Journal of Biological Chemistry 291, no. 16: 8816–8824. 10.1074/jbc.M116.718460. 26896795 PMC4861449

[fsn371498-bib-0054] Zhang, D. , Y. P. Jian , Y. N. Zhang , et al. 2023. “Short‐Chain Fatty Acids in Diseases.” Cell Communication and Signaling: CCS 21, no. 1: 212. 10.1186/s12964-023-01219-9. 37596634 PMC10436623

[fsn371498-bib-0055] Zhang, W. Q. , T. T. Zhao , D. K. Gui , et al. 2019. “Sodium Butyrate Improves Liver Glycogen Metabolism in Type 2 Diabetes Mellitus.” Journal of Agricultural and Food Chemistry 67, no. 27: 7694–7705. 10.1021/acs.jafc.9b02083. 31250637

[fsn371498-bib-0061] Zhang, X. , C. C. Olabisi Oluwabukola , and S. H. Eagle 2021b. “Dietary Cholesterol Drives Fatty Liver‐Associated Liver Cancer by Modulating Gut Microbiota and Metabolites.” Gut 70, no. 4: 761–774. 10.1136/gutjnl-2019-319664. 32694178 PMC7948195

[fsn371498-bib-0056] Zhang, X. , Y. Zhang , M. Zhou , et al. 2021a. “DPHC From *Alpinia officinarum* Ameliorates Oxidative Stress and Insulin Resistance via Activation of Nrf2/ARE Pathway in db/db Mice and High Glucose‐Treated HepG2 Cells.” Frontiers in Pharmacology 12: 792977. 10.3389/fphar.2021.792977. 35111058 PMC8801804

[fsn371498-bib-0057] Zhou, N. , X. Gu , T. Zhuang , Y. Xu , L. Yang , and M. Zhou . 2020. “Gut Microbiota: A Pivotal Hub for Polyphenols as Antidepressants.” Journal of Agricultural and Food Chemistry 68, no. 22: 6007–6020. 10.1021/acs.jafc.0c01461. 32394713

[fsn371498-bib-0058] Zhou, Y. , L. Liu , R. Xiang , et al. 2023. “Arctigenin Mitigates Insulin Resistance by Modulating the IRS2/GLUT4 Pathway via TLR4 in Type 2 Diabetes Mellitus Mice.” International Immunopharmacology 114: 109529. 10.1016/j.intimp.2022.109529. 36481528

[fsn371498-bib-0059] Zhu, X. , D. Zhang , Y. Wang , C. Wang , X. Liu , and Y. Niu . 2024. “Study on the Signaling Pathways Involved in the Anti‐Hyperglycemic Effect of Raspberry Ketone on Zebrafish Using Integrative Transcriptome and Metabolome Analyses.” Food & Function 15, no. 18: 9457–9470. 10.1039/d4fo01675k. 39189875

[fsn371498-bib-0060] Zhu, Y. , B. Chen , X. Zhang , et al. 2024. “Exploration of the *Muribaculaceae* Family in the Gut Microbiota: Diversity, Metabolism, and Function.” Nutrients 16, no. 16: 2660. 10.3390/nu16162660. 39203797 PMC11356848

