# Supplementary material for: Wild Prunus cerasifera Ehrh. Polyphenols Alleviate Hyperglycemia in Type 2 Diabetes Mellitus Mice via Modulating Gut Microbiota‐SCFAs‐PI3K/Akt/TBC1D4 Pathway
Source: Food Sci Nutr. 2026 Feb 8;14(2):e71498. doi: 10.1002/fsn3.71498 (PMC12883564; doi:10.1002/fsn3.71498)
Supplement: Supplementary file 1 — Appendix S1: fsn371498‐sup‐0001‐AppendixS1.docx. [file FSN3-14-e71498-s001.docx]

**Supplementary Materials**

1. **Supplementary experimental method**

**1.1 Determination of total sugar**

The total sugar content was determined based on phenol-sulfuric acid method, 1.0 mL of 6.0% phenol was added to 2.0 mL of sample solution. Then, 5.0 mL of concentrated sulfuric acid was mixed slowly, and allowed to react at room temperature for 30 minutes. A blank was prepared by substituting the sample with 1.0 mL of distilled water. The absorbance was measured at 490 nm. The total sugar concentration in sample was calculated based on the curve formula plotted with glucose (20-100 μg/mL) as standard (y = 228.41x - 8.8438, R^2^=0.9947).

**1.2 Determination of total proteins**

The total proteins content was determined using the Coomassie Brilliant Blue G-250 method. Briefly, 0.1 mL of sample solution was mixed with 5.0 mL of Coomassie Brilliant Blue reagent and incubated at 25 ^o^C for 5 min. Absorbance was measured at 595 nm, and protein concentration was calculated based on the curve formula plotted with bovine serum albumin (BSA) (50-500 μg/mL) as standard (y = 3.5604x - 0.2737, R^2^=0.9933) standard.

**1.3 Determination of ash content**

The ash content was determined by incineration in a muffle furnace. Briefly, samples were weighed into pre-ashed crucibles and heated at 550 °C until a constant weight was obtained. After cooling in a desiccator, the ash content was calculated as the percentage of residue relative to the initial sample weight.

1. **Supplemental results**


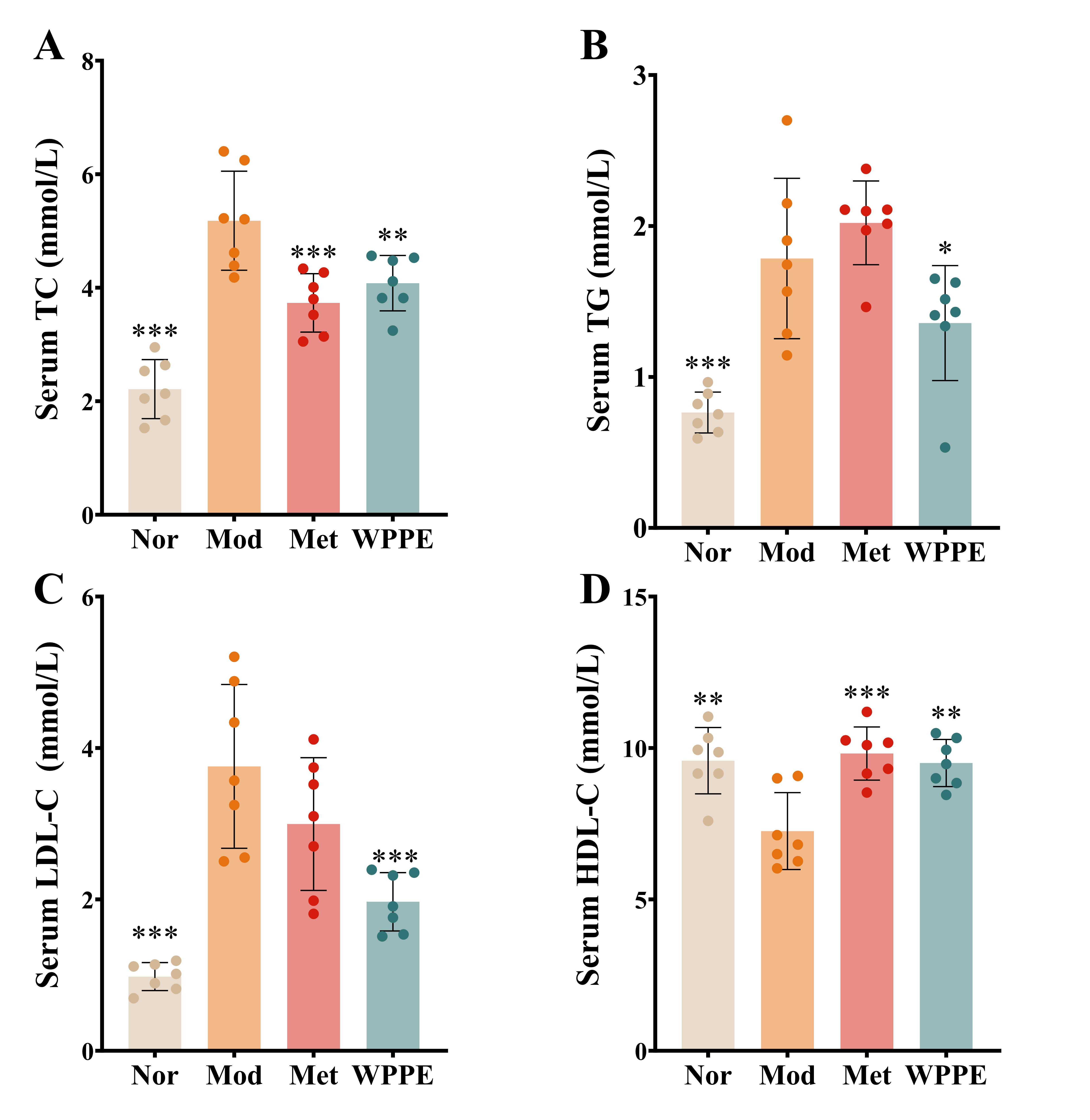


Fig. S1 Effect of WPPE on serum TC (A), TG (B), LDL-C (C) and HDL-C (D) in mice.





Fig S2 Rarefaction curves of gut microbiota in different groups

Table S1 The detection limits (LOD/LOQ) and source information of biochemical assay kits

| Parameter | LOD | LOQ | Product Number | Company |
| --- | --- | --- | --- | --- |
| TC | 0 mmol/L | 19.39 mmol/L | A111-1-1 | Jiancheng Bioengineering Institute |
| TG | 0.3 mmol/L | 11.4 mmol/L | A110-1-1 |  |
| Hb1A | 1.33/g prot | 23.5/g prot | A056-1-1 |  |
| GSP | 0 mmol/L | 100 mmol/L | A037-2-1 |  |
| AKP | 0 U/L | 190 U/L | A059-2 |  |
| AST | 0 U/L | 190 U/L | C010-2-1 |  |
| ALT | 0 U/L | 200 U/L | C009-2-1 |  |
| SOD | 0.5 U/mL | 100 U/L | A001-3 |  |
| MDA | 0 nmol/mg prot | 100 nmol/mg prot | A003-1 |  |
| CAT | 0.113 (OD_405nm_) | 0.651 (OD_405nm_) | A007-1-1 |  |
| TNF-α | 30.8 pg/mL |  | PT512 | Biyuntian Biotechnology |
| IL-6 | 12.8 pg/ mL |  | PI326 |  |
| IL-4 | 14.5 pg/ mL |  | PI612 |  |

Table S2 The content of total phenolics, flavonoids, sugar, proteins and ash in WPPE

|  | Total phenolics  (mg GAE/g E) | Total flavonoids  (mg QuE/g E) | Total sugar  (mg GLU/g E) | Total proteins  (mg BSA/g E) | Ash content  (%) |
| --- | --- | --- | --- | --- | --- |
| WPPE | 219.27 ± 1.57 | 165.58 ± 9.14 | 326.61 ± 18.53 | 75.20 ± 5.33 | 0.34 ± 0.11 |

Table S3 RT-qPCR amplification primers of hepatic differential genes

| Genetics | Sequence (5' to 3') |
| --- | --- |
| *Pi3k*-F | CTGGAATGTGTGGCTGGAGT |
| *Pi3k*-R | AGGAGGAAGCGGTGGTCTAT |
| *Akt*-F | ATGAACGACGTAGCCATTGTG |
| *Akt*-R | TTGTAGCCAATAAAGGTGCCAT |
| *Gapdh*-F | AGGTCGGTGTGAACGGATTTG |
| *Gapdh*-R | TGTAGACCATGTAGTTGAGGTCA |
| *Tbc1d4*-F | CCAACAGTCTTGCCTCAGAG |
| *Tbc1d4*-R | GAATGTGTGAGCCCGTCTTC |
| *Gck*-F | TGAGCCGGATGCAGAAGGA |
| *Gck*-R | GCAACATCTTTACACTGGCCT |
| *Glut2*-F | GCCTGTGTATGCAACCATTG |
| *Glut2*-R | TGGCCCAATCTCAAAGAAAC |

Table S4 The standard curves of SCFAs

| Sample | Calibration curves | R² | Linear range |
| --- | --- | --- | --- |
| Acetic acid | Y = (X - 4.1297)/369.89 | 0.9958 | 0.01<Y<1 mg/mL |
| Propionic acid | Y = (X+6.4555)/656.49 | 0.9954 | 0.01<Y<1 mg/mL |
| Isobutyric acid | Y = (X+6.2722)/975.03 | 0.9959 | 0.01<Y<1 mg/mL |
| Butyric acid | Y = (X-10.862)/887.88 | 0.997 | 0.01<Y<1 mg/mL |
| Isovaleric acid | Y = (X+6.165)/1125.5 | 0.9927 | 0.01<Y<1 mg/mL |
| Valeric acid | Y = (X+7.6594)/1170.9 | 0.9933 | 0.01<Y<1 mg/mL |

Table S5 The p-value correction coefficients among biochemical parameters, top 29 gut microbiota and key genes relating with glucose metabolism (n=5)

|  | **Acetic acid** | | **AKP** | **ALT** | **AST** | **Butyric acid** | **CAT** | **IL-4** | **IL-6** | **Isobutyric acid** | **Isovaleric acid** | **MDA** | **Propanoic acid** | **SOD** | **TC** | **TG** | **TNF-α** |
| --- | --- | --- | --- | --- | --- | --- | --- | --- | --- | --- | --- | --- | --- | --- | --- | --- | --- |
| *Akt* | | 0.0602 | 0.0000 | 0.0069 | 0.0055 | 0.1615 | 0.0123 | 0.2212 | 0.1297 | 0.0759 | 0.0187 | 0.3074 | 0.0005 | 0.0204 | 0.7435 | 0.0046 | 0.0696 |
| *Alistipes* | | 0.6389 | 0.2110 | 0.0915 | 0.4343 | 0.8297 | 0.6669 | 0.2160 | 0.4266 | 0.7241 | 0.2212 | 0.6575 | 0.0946 | 0.0454 | 0.5067 | 0.3334 | 0.2478 |
| *Bacteroides* | | 0.8324 | 0.2160 | 0.3607 | 0.3677 | 0.6615 | 0.4657 | 0.6206 | 0.7532 | 0.6389 | 0.8124 | 0.5151 | 0.0369 | 0.2479 | 0.2424 | 0.6858 | 0.5281 |
| *Bifidobacterium* | | 1.0000 | 0.2563 | 0.0232 | 0.2739 | 0.4404 | 0.1090 | 0.8149 | 0.0253 | 0.7563 | 0.6661 | 0.4704 | 0.1365 | 0.8001 | 0.0089 | 0.3340 | 0.4080 |
| *Blautia* | | 0.6615 | 0.4235 | 0.8198 | 0.4350 | 0.9344 | 0.1156 | 0.9144 | 0.5197 | 0.3973 | 0.8694 | 0.2829 | 0.4981 | 0.0836 | 0.9094 | 0.2260 | 0.5427 |
| *Candidatus_Saccharimonas* | | 0.2563 | 0.6430 | 0.1838 | 0.8297 | 0.4780 | 0.7563 | 0.9044 | 0.0673 | 0.1411 | 0.1451 | 0.1637 | 0.6890 | 0.6157 | 0.0105 | 0.5978 | 0.4423 |
| *Clostridia_vadinBB60_group* | | 0.9234 | 0.4343 | 0.1779 | 0.4343 | 0.2829 | 0.4657 | 0.1094 | 0.8929 | 0.5493 | 0.5756 | 0.3892 | 0.2152 | 0.3966 | 0.0602 | 0.1525 | 0.5538 |
| *Clostridium_sensu_stricto_1* | | 0.8792 | 0.1548 | 0.1276 | 0.2646 | 0.3764 | 0.1528 | 0.6658 | 0.0670 | 0.5151 | 0.8792 | 0.8196 | 0.1561 | 0.7706 | 0.0083 | 0.2101 | 0.7342 |
| *Colidextribacter* | | 0.2479 | 0.6669 | 0.5580 | 0.5067 | 0.3510 | 0.8425 | 0.9132 | 0.1094 | 0.3203 | 0.9847 | 0.4901 | 0.5405 | 0.2012 | 0.7827 | 0.5235 | 0.4388 |
| *Coriobacteriaceae_UCG-002* | | 0.1637 | 0.6248 | 0.6708 | 0.6293 | 0.8643 | 0.8346 | 0.2921 | 0.0010 | 0.1106 | 0.1573 | 0.4989 | 0.8567 | 0.0451 | 0.0308 | 0.5625 | 0.0066 |
| *Desulfovibrio* | | 0.0058 | 0.4738 | 0.6763 | 0.2479 | 0.0809 | 0.7144 | 0.7926 | 0.0666 | 0.0052 | 0.0117 | 0.2949 | 0.3369 | 0.1408 | 0.0213 | 0.6206 | 0.1594 |
| *Desulfovibrionaceae* | | 0.2316 | 0.2264 | 0.5493 | 0.2160 | 0.7903 | 0.3011 | 0.4190 | 0.0000 | 0.1607 | 0.0422 | 0.0363 | 0.5193 | 0.0007 | 0.0253 | 0.0944 | 0.0140 |
| *Dubosiella* | | 0.9132 | 0.0944 | 0.2708 | 0.4420 | 0.1956 | 0.0834 | 0.5580 | 0.5493 | 0.8425 | 0.5151 | 0.6763 | 0.0917 | 0.3011 | 0.2012 | 0.4578 | 0.4784 |
| *Enterorhabdus* | | 0.9950 | 0.6895 | 0.6339 | 0.5197 | 0.0991 | 0.2563 | 0.1769 | 0.6754 | 0.5668 | 0.9295 | 0.2769 | 0.4478 | 0.8544 | 0.8994 | 0.9446 | 0.1731 |
| *Faecalibaculum* | | 0.2708 | 0.3203 | 0.4266 | 0.5934 | 0.8794 | 0.1446 | 1.0000 | 0.1192 | 0.2479 | 0.3011 | 0.9949 | 0.4862 | 0.5844 | 0.0454 | 0.4420 | 0.2535 |
| *Gck* | | 0.0711 | 0.0000 | 0.0001 | 0.0002 | 0.0809 | 0.0005 | 0.7630 | 0.3538 | 0.0275 | 0.0644 | 0.1779 | 0.0000 | 0.0392 | 0.7144 | 0.0052 | 0.2423 |
| *Glut2* | | 0.1446 | 0.0055 | 0.0562 | 0.0049 | 0.0192 | 0.0014 | 0.1333 | 0.4190 | 0.2592 | 0.0834 | 0.0438 | 0.0169 | 0.0275 | 0.7048 | 0.0077 | 0.5030 |
| *Helicobacter* | | 0.7048 | 0.0286 | 0.0117 | 0.1370 | 0.3273 | 0.0666 | 0.5320 | 0.7241 | 0.4901 | 0.1094 | 0.8727 | 0.0037 | 0.2708 | 0.5756 | 0.1032 | 0.2507 |
| *Ileibacterium* | | 0.5406 | 0.3074 | 0.0488 | 0.5320 | 0.6989 | 0.2264 | 0.9540 | 0.0438 | 0.3966 | 0.4901 | 0.3074 | 0.2101 | 0.9949 | 0.0031 | 0.3401 | 0.4466 |
| *Lachnospiraceae* | | 0.5756 | 0.2887 | 0.0543 | 0.4039 | 0.5800 | 0.1297 | 0.6858 | 0.0915 | 0.4420 | 0.6389 | 0.5067 | 0.2363 | 0.9949 | 0.0069 | 0.3138 | 0.8594 |
| *Lachnospiraceae_NK4A136* | | 0.0688 | 0.9234 | 0.2649 | 0.9336 | 0.8198 | 0.4984 | 0.4420 | 0.0123 | 0.0543 | 0.0834 | 0.5067 | 0.9093 | 0.4266 | 0.0041 | 0.8224 | 0.0617 |
| *Lactobacillus* | | 0.2767 | 0.0015 | 0.0666 | 0.0506 | 0.5239 | 0.0156 | 0.3334 | 0.0117 | 0.1485 | 0.0488 | 0.1735 | 0.0215 | 0.0004 | 0.4039 | 0.0179 | 0.0564 |
| *Ligilactobacillus* | | 0.1159 | 0.0195 | 0.1063 | 0.0688 | 0.5030 | 0.2160 | 0.1063 | 0.0086 | 0.1824 | 0.0187 | 0.4190 | 0.0333 | 0.0735 | 0.5406 | 0.0407 | 0.0004 |
| *Limosilactobacillus* | | 0.1032 | 0.0407 | 0.2708 | 0.0666 | 0.2650 | 0.1607 | 0.2949 | 0.0000 | 0.0562 | 0.0069 | 0.0336 | 0.0833 | 0.0025 | 0.0543 | 0.0809 | 0.0159 |
| *Mucispirillum* | | 0.3677 | 0.0471 | 0.0454 | 0.0666 | 0.9446 | 0.0298 | 0.6763 | 0.0377 | 0.1870 | 0.0735 | 0.1297 | 0.0415 | 0.1094 | 0.2708 | 0.0438 | 0.0289 |
| *Muribaculaceae* | | 0.5844 | 0.2110 | 0.1227 | 0.2535 | 0.4545 | 0.2160 | 0.9540 | 0.0454 | 0.4266 | 0.7337 | 0.5067 | 0.1038 | 0.8124 | 0.0062 | 0.3268 | 0.5155 |
| *Odoribacter* | | 0.1094 | 0.0095 | 0.0363 | 0.0163 | 0.0878 | 0.0195 | 0.0783 | 0.0783 | 0.1691 | 0.0666 | 0.0179 | 0.0122 | 0.0086 | 0.8929 | 0.0027 | 0.0628 |
| *Oscillospiraceae* | | 0.5320 | 0.2370 | 0.0195 | 0.2708 | 0.2478 | 0.0562 | 0.5320 | 0.4738 | 0.5235 | 0.9132 | 0.3966 | 0.1388 | 0.6669 | 0.0759 | 0.0888 | 0.7708 |
| *Pi3k* | | 0.3074 | 0.0735 | 0.2592 | 0.0524 | 0.2423 | 0.0944 | 0.6763 | 0.0002 | 0.1735 | 0.0349 | 0.0018 | 0.1022 | 0.0488 | 0.1094 | 0.0232 | 0.0099 |
| *Rikenella* | | 0.8994 | 0.9647 | 0.2983 | 0.9899 | 0.7926 | 0.5625 | 0.5113 | 0.3649 | 0.8994 | 0.4866 | 0.4388 | 0.6241 | 0.4311 | 0.9194 | 0.6615 | 0.3574 |
| *Rikenellaceae_RC9* | | 0.2563 | 0.1932 | 0.6293 | 0.2395 | 0.5863 | 0.5155 | 0.2105 | 0.0082 | 0.1981 | 0.0188 | 0.1106 | 0.1888 | 0.0033 | 0.1243 | 0.1658 | 0.1086 |
| *Romboutsia* | | 0.3326 | 0.8891 | 0.6746 | 0.7800 | 0.8839 | 0.6934 | 0.7508 | 0.0193 | 0.1419 | 0.3671 | 0.2637 | 0.6278 | 0.4108 | 0.0004 | 0.8891 | 0.7026 |
| *Ruminococcaceae* | | 0.8544 | 0.1073 | 0.0426 | 0.2860 | 0.8842 | 0.0426 | 0.9697 | 0.2739 | 0.8297 | 0.2983 | 0.1261 | 0.2122 | 0.1861 | 0.9094 | 0.0534 | 0.4155 |
| *Tbc1d4* | | 0.0223 | 0.0000 | 0.0007 | 0.0003 | 0.0108 | 0.0001 | 0.5235 | 0.1824 | 0.0135 | 0.0243 | 0.1094 | 0.0000 | 0.0058 | 0.9744 | 0.0003 | 0.0595 |

Table S6 The p-value correction coefficients among top 29 gut microbiota and key genes relating with glucose metabolism (n=5)

|  | ***Akt*** | ***Gck*** | ***Glut2*** | ***Pi3k*** | ***Tbc1d4*** |
| --- | --- | --- | --- | --- | --- |
| *Alistipes* | 0.7337 | 0.9744 | 0.5580 | 0.5151 | 0.9642 |
| *Bacteroides* | 0.4901 | 0.4190 | 0.0944 | 0.2012 | 0.7532 |
| *Bifidobacterium* | 0.0000 | 0.0008 | 0.0043 | 0.2314 | 0.0000 |
| *Blautia* | 0.8944 | 0.8994 | 0.2860 | 0.4989 | 0.8644 |
| *Candidatus_Saccharimonas* | 0.0009 | 0.0049 | 0.0515 | 0.4704 | 0.0075 |
| *Clostridia_vadinBB60_group* | 0.4114 | 0.6206 | 0.3401 | 0.9031 | 0.4114 |
| *Clostridium_sensu_stricto_1* | 0.0027 | 0.0000 | 0.0104 | 0.1119 | 0.0000 |
| *Colidextribacter* | 0.7435 | 0.4819 | 0.7926 | 0.6669 | 0.2708 |
| *Coriobacteriaceae_UCG-002* | 0.0221 | 0.0117 | 0.0650 | 0.9647 | 0.0055 |
| *Desulfovibrio* | 0.1227 | 0.0058 | 0.1297 | 0.6575 | 0.0471 |
| *Desulfovibrionaceae* | 0.1032 | 0.1063 | 0.0888 | 0.3138 | 0.0582 |
| *Dubosiella* | 0.6669 | 0.1063 | 0.0711 | 0.2949 | 0.0915 |
| *Enterorhabdus* | 0.9094 | 0.9950 | 0.7466 | 0.7322 | 0.7660 |
| *Faecalibaculum* | 0.0363 | 0.0000 | 0.0187 | 0.3011 | 0.0001 |
| *Helicobacter* | 0.1525 | 0.0623 | 0.0077 | 0.0232 | 0.0644 |
| *Ileibacterium* | 0.0002 | 0.0018 | 0.0007 | 0.1963 | 0.0006 |
| *Lachnospiraceae* | 0.0003 | 0.0000 | 0.0156 | 0.1408 | 0.0013 |
| *Lachnospiraceae_NK4A136_group* | 0.0009 | 0.0038 | 0.0253 | 0.6575 | 0.0055 |
| *Lactobacillus* | 0.7048 | 0.5756 | 0.5934 | 0.0232 | 0.7048 |
| *Ligilactobacillus* | 0.7728 | 0.7532 | 0.9744 | 0.2316 | 0.8324 |
| *Limosilactobacillus* | 0.0944 | 0.2012 | 0.1870 | 0.2592 | 0.1691 |
| *Mucispirillum* | 0.9234 | 0.4190 | 0.4984 | 0.1824 | 0.4578 |
| *Muribaculaceae* | 0.0004 | 0.0005 | 0.0073 | 0.0562 | 0.0003 |
| *Odoribacter* | 0.9540 | 0.9744 | 0.5844 | 0.3607 | 0.6206 |
| *Oscillospiraceae* | 0.0095 | 0.0223 | 0.0129 | 0.1607 | 0.0123 |
| *Rikenella* | 0.4388 | 0.8297 | 0.8247 | 0.6801 | 0.5538 |
| *Rikenellaceae_RC9_gut_group* | 0.1073 | 0.0564 | 0.5030 | 0.3015 | 0.1471 |
| *Romboutsia* | 0.0049 | 0.0002 | 0.1268 | 0.4813 | 0.0094 |
| *Ruminococcaceae* | 0.6293 | 0.1747 | 0.2709 | 0.3719 | 0.1615 |
